# The role of N185D substitution in enhancing activity of BaqA∆C α-amylase

**DOI:** 10.1038/s41598-025-27398-8

**Published:** 2025-12-05

**Authors:** Muhammad Aqib Hanif, Fernita Puspasari, Handajaya Rusli, Reza Aditama, Ihsanawati Ihsanawati, Keni Vidilaseris, Dessy Natalia

**Affiliations:** 1 Biochemistry and Biomolecular Engineering Research Group, Faculty of Mathematics and Natural Sciences, Institut Teknologi Bandung, Jl. Ganesha No. 10, Bandung, Indonesia; 2 Analytical Chemistry Research Group, Faculty of Mathematics and Natural Sciences, Institut Teknologi Bandung, Jl. Ganesha No. 10, Bandung, Indonesia; 3 The Molecular and Integrative Biosciences Research Programme (MIBS), Faculty of Biological and Environmental Sciences, University of Helsinki, Biocenter 1/Viikinkaari 9, Helsinki, 00014 Finland

**Keywords:** α–amylase, BaqA, BaqA∆C, CSRV, N185D, Biochemistry, Biotechnology

## Abstract

α-Amylases are endoenzymes capable of degrading starch into branched and linear oligosaccharides. Since starch-processing industries rely on starch-rich mashes, exploring amylases that can efficiently hydrolyse highly concentrated raw starch is crucial. BaqA from *Bacillus aquimaris* MKSC 6.2 is particularly promising as it hydrolyses raw starch into simple sugars without preheating, offering potential energy and cost savings for industrial applications. This study investigates the functional role of a specific residue within Conserved Sequence Region (CSR) V of BaqAΔC (a truncated BaqA lacking 34 amino acids from the C terminus), predicted to influence catalytic activity, product specificity, and substrate binding. A BaqA∆CN185D mutant was generated through site-directed mutagenesis and expressed in *Escherichia coli* ArcticExpress (DE3). Compared with BaqAΔC, the mutant displayed higher specific activity (7.6 ± 0.2 vs. 5.8 ± 0.4 U/mg) and a greater degree of hydrolysis (2.18% vs. 1.41%). Futhermore, BaqAΔCN185D showed a significantly lower *K*_*m*_ (9.48 ± 1.3 mg/mL) compared to BaqAΔC (23.48 ± 4.8 mg/mL), reflecting higher substrate affinity. Its catalytic efficiency (*k*_*ca.*t_/*K*_*m*_) also increased from 5.65 ± 0.5 to 9.8 ± 0.9 mL·mg⁻¹·s⁻¹, indicating enhanced substrate binding and overall catalytic performance due to the N185D mutation. HPLC analysis of crude protein extracts from *E. coli* ArcticExpress (DE3) expressing BaqA∆C and BaqA∆CN185D revealed saccharifying activity, whereas the control strain produced a native liquefying α-amylase. Molecular dynamics simulations further indicated that the N185D substitution altered catalytic and substrate-binding site residues, likely creating a more effective substrate-binding gateway. Collectively, these findings highlight the critical role of N185 in modulating BaqA∆C’s catalytic efficiency and product profile, providing valuable insights for industrial enzyme engineering.

**Supplementary Information:**

The online version contains supplementary material available at 10.1038/s41598-025-27398-8.

## Introduction

 α-Amylases (EC 3.2.1.1) are the endoenzymes belonging to the glycoside hydrolase (GH) 13 family of enzymes having the ability to catalyse the *α*−1,4-glucosidic linkages in starch while retaining the α-anomeric configuration mechanism of reaction^1,2^. According to the sequence-based classification scheme of the Carbohydrate-Active enzymes database (CAZy; http://www.cazy.org/)^3,4^, α-amylases constitute the largest group of Glycoside Hydrolases (GHs)^5^. The GH13 family comprises 49 subfamilies, though only 17 exhibit specific α-amylase activity^4,6–8^. Three-dimensional (3D) structures revealed that α-amylases are monomeric enzymes with a single polypeptide chain folded into three domains (A, B, and C). Domain A features a (β/α)8-barrel^1^ with the catalytic residues consisting of aspartate (nucleophile), glutamate (proton donor) and aspartate (transition state stabiliser). Domain B, located between the third ß-sheet and adjacent α-helix, is involved in Ca^2+^ binding (due to the presence of a Ca^2+^ binding site in the interface of domains A and B) or substrate binding^9,10^, and differs in size and structure among species^11^. Some α-amylases, such as *Geoacillus thermoleovorans*, also possess a chloride ion binding site in the active site to improve the catalytic efficiency, most likely by raising the pKa of the hydrogen-donating residue in the active site^12^. Domain C folds into 8 antiparallel ß-strands and in some cases thought to contribute in stabilising the catalytic domain by shielding the hydrophobic residues within the domain A from solvent exposure^13^.

According to Cihan et al. (2018), secondary structure analysis of various α-amylases has provided insights about some residues that may be associated with sugar-binding pockets. Although these findings have not been implicated in previous studies, they have proven valuable for more in-depth structural investigations. It has been proposed that several conserved sequence regions (CSRs) are essential for enzymatic activity in maltose-binding residues found in enzymes like GTA and TASKA^14^. The conserved areas CSR II, III and IV seem to be crucial for substrate binding as well as catalytic activity. In contrast, the CSR VI, I, V, and VII are likely involved in binding with substrate and enzyme specificity within these subfamilies^2,12,15,16^. Investigating conserved residues within enzyme subfamilies offers a strategic approach to modifying catalytic properties, as these residues are closely linked to the enzyme specificity of that particular subfamily^17^.

BaqA, an α-amylase isolated from *Bacillus aquimaris* MKSC 6.2, (recently reclassified as *Rosellomorea aquimaris*), which is a soft coral-associated marine bacterium from the island of Merak Kecil, Banten, Indonesia, exhibits considerable potential for industrial applications^18^. One of its most significant advantages is its capacity to hydrolyze raw starch directly into oligosaccharides without requiring preheating. This characteristic makes BaqA highly desirable for energy-efficient bioprocessing since it provides a significant decrease in energy usage and total production costs^18^. A conserved LPDlx motif in the CSR-V region, a unique aromatic motif at the C-terminus, and a unique pair of tryptophan residues (Trp201 and Trp202, according to BaqA numbering) situated within the α3 helix of the catalytic TIM-barrel domain are among the structural features of BaqA that distinguish it as belonging to the glycoside hydrolase family 13 subfamily 45 (GH13_45)^19^. Furthermore, a truncated variant, BaqA∆C (lacking 34 amino acids from the C terminus), demonstrates improved solubility, stability and halotolerance, making it particularly suitable for use in starch processing industries^20^.

The CSR-V region in α-amylases is predicted to function as a binding pocket, influencing the substrate binding and enzyme activity. Comparative analysis of the CSRs revealed that some residues in BaqA∆C are not conserved when compared to other members of GH13_45 subfamily. Specifically, in CSR V, BaqA∆C contains asparagine (residue 185), whereas most α-amylases have alanine at this position^14^. In ASKA, alanine is well conserved at this identical position (161 as ASKA numbering). A previous study reported a single-point mutation at position 161, where alanine was substituted with aspartate. This mutation resulted in enhanced enzymatic activity, increased thermostability and more production of maltose as the end product^21^. Given its predicted involvement in substrate binding and potential contribution to stability, CSR-V was selected for targeted investigation in this study. To evaluate its functional importance, site-directed mutagenesis was conducted on recombinant BaqAΔC, targeting a specific residue inside CSR-V. Particularly, the asparagine at position 185 (N185, BaqAΔC numbering) was substituted with aspartic acid. This substitution was intended to imitate the conserved characteristics present in analogous enzymes and to assess the influence of this residue on enzymatic characteristics like substrate binding, catalytic efficiency, structural stability, and product selectivity.

## Materials and methods

### Bioinformatics analysis

A total of 140 amino acid sequences, proposed by^22^ to represent a novel GH13_45 subfamily and exemplified by BaqA (BaqA accession no. JN797599.1), were retrieved from the GenBank database^23^. The sequences were analysed based on the polypeptide chain covering CSR-I (strand β3) and CSR-II (strand β4), including the entire domain B, the two consecutive tryptophan residues in helix α3 of the catalytic TIM-barrel and the specific sequence in CSR-V positioned in domain B. Sequence alignments were performed using Clustal-Omega available on the European Bioinformatics Institute’s server (http://www.ebi.ac.uk/) and BioEdit^24^. A web logo for their seven CSRs was generated using the WebLogo 3.0 server (http://weblogo.berkeley.edu/). The three-dimensional structure of BaqAΔC and BaqA∆CN185D was modelled by I-TASSER (Iterative Threading ASSEmbly Refinement)^25^ and Alphafold2^26^, using the X-ray crystal structure of *G. thermoleovorans* α-amylase (PDB code: 4E2O) as a template. All 3D structures were visualized by PyMOL^27^.

### Strains, plasmids, and chemicals

The recombinant plasmid pET-30a-BaqA∆C was obtained from the plasmid collection at the Biochemistry and Biomolecular Research Group, Faculty of Mathematics and Natural Sciences, Institut Teknologi Bandung. *E. coli* TOP10F’ (Thermo Scientific, USA) and *E. coli* ArcticExpress (DE3) (Stratagene, USA) were used for cloning and expression, respectively. *Pfu* Turbo DNA polymerase and *DpnI* (Thermo Scientific, USA) were used for repetitive PCR and molecular cloning experiments. Plasmids were isolated with the Presto™ Mini Plasmid Kit (Geneaid, Taiwan). 1 kb DNA ladder and protein marker (PM2700) were obtained from SMOBIO Technology, Taiwan. Nickel-NTA agarose resin was obtained from Qiagen (Netherlands). Protein quantification and electrophoresis kits were obtained from HIMEDIA, India. Raw and soluble starches for activity assay were obtained from Merck, USA.

### Site-directed mutagenesis

The BaqAΔCN185D mutant was constructed by site-directed mutagenesis using pET30a-BaqAΔC^20^ as a template. PCR was performed with *Pfu* Turbo DNA polymerase (Thermo Scientific, USA) using primers N185D-F 5′- GGC CTT CCT GAT TTA GAC ACA GAG AAT CCC GAA − 3′ and N185D-R 5′- TTC GGG ATT CTC TGT GTC TAA ATC AGG AAG GCC − 3’, synthesized by Macrogen (Singapore). The reaction conditions were as follows: initial denaturation at 95 °C for 5 min, followed by 16 cycles of denaturation at 95 °C for 1 min, annealing at 46.5 °C for 1 min, extension at 72 °C for 1.5 min, and a final extension at 72 °C for 1 min. PCR products were digested with *DpnI* (Thermo Scientific, USA) at 37 °C for 2 h to remove template DNA. The resulting plasmid was then transformed into *E. coli* TOP10F’ competent cells, and positive clones were confirmed by Sanger sequencing (Macrogen, Singapore).

### Expression and purification

The pET30a-BaqAΔC plasmid was transformed into *E. coli* ArticExpress (DE3) using the heat-shock method. Transformants were grown for 12–16 h in Luria Bertani (LB) medium (0.5% yeast extract, 1% tryptone, and 1% NaCl) supplemented with kanamycin (50 µg/mL) in a shaker incubator at 37 °C, 150 rpm. The culture (1.5% v/v) was inoculated into 100 mL fresh LB medium containing kanamycin (50 µg/mL) and incubated until OD600 reached 0.6–0.8. Expression was induced by the addition of 0.25 mM isopropyl-β-D-1-thiogalactopyranoside (IPTG), followed by incubation at 10 °C for 24 h, 150 rpm. Cells were harvested by centrifugation at 5000 rpm for 10 min at 4 °C, washed with 5 mL of ddH_2_O and centrifuged again under the same conditions.

Cell pellets were resuspended in lysis buffer (50 mM HEPES pH 8, 500 mM NaCl, and 10 mM imidazole) containing protease inhibitor cocktail (1:4, w/v) and disrupted by sonication for 15 min at 4 °C (30 kHz, 30 s on/off cycles). Lysates were clarified by centrifugation at 12,000 x g for 15 min at 4 °C. Protein expression was analysed by SDS-PAGE, and protein concentration was determined by the Bradford method.

His-tagged proteins were purified by Ni-NTA affinity chromatography. Crude extracts were incubated with Ni-NTA resin overnight at 4 °C with gentle rotation. Unbound materials were collected manually as a flow-through fraction. The resin was washed using 15 column volumes (CVs) of wash buffer (50 mM HEPES pH 8, 500 mM NaCl, and 20 mM imidazole), and proteins were eluted sequentially with Elution Buffer 1–4 (with varying concentrations of imidazole from 50 to 150mM). Imidazole was removed by ultrafiltration (Vivaspin^®^ Turbo 4, 10 kDa MWCO) and buffer-exchanged into 50 mM HEPES pH 8.0. The purified protein was used for subsequent biochemical analyses.

### α-Amylase activity assay

Starch-hydrolysing activity of BaqAΔC and BaqAΔCN185D was measured using the 3,5-dinitrosalicylic acid (DNS) method^28,29^. Reaction mixtures contained 25 µL of the purified enzyme and 25 µL of 2% (w/v) soluble starch in a universal buffer (25 mM MES pH 6.5, 50 mM Malate, and 50 mM Tris). Reactions were incubated at 50 °C for 15 min and terminated by adding 50 µL of DNS solution (1% (w/v), 20% (v/v) 2 M NaOH, and 30% (w/v) KNa-tartaric). Samples were boiled for 10 min, diluted with ddH_2_O to a final volume of 1 mL, and absorbance was measured at 500 nm. A glucose standard curve was used to calculate the amount of reducing sugar. All reactions were measured in triplicate. One unit of enzyme activity was defined as the amount of enzyme required to produce 1 µmol of glucose per minute under the assay conditions.

### Effect of temperature and pH on the activity of BaqAΔC and BaqAΔCN185D

The effect of pH on the hydrolysis of soluble starch was determined using 2% (w/v) soluble starch in universal Malate buffer across pH 4–8.5. Reactions were incubated at 50 °C for 15 min. The effect of temperature was measured by incubating reaction mixtures at 30–80 °C. Enzyme stability was determined by pre-incubating the enzyme at 50 °C for 15, 30, 60, 120, 240, and 360 min, followed by measurement of residual activity. The amount of reducing sugar was determined by the DNS method.

### Determination of kinetic parameters of BaqAΔC and BaqAΔCN185D

Kinetic parameters of BaqAΔC and BaqAΔCN185D were determined at the optimum assay conditions by varying the concentration of soluble starch from 10 to 45 mg/mL in 50 mM universal buffer (pH 6.5). Reducing sugar formed during the assay was measured following the DNS method described above. The kinetic parameter values (*V*_*max*_, *k*_*cat*_, *K*_*m*_, and *k*_*cat*_*/K*_*m*_) were obtained by fitting the initial reaction rate to the Michaelis-Menten equation. Fittings and calculations were performed using the GraphPad Prism version 10.0 program for windows^30^.

### Effect of EDTA and [Ca^2+^] on activity of BaqAΔC and BaqAΔCN185D

The effect of EDTA and [Ca^2+^] was examined using the previous DNS assay. Enzyme mixture was pre-incubated with various concentrations (1, 3, 5, 7 and 10 mM) of EDTA and [Ca^2+^]. Moreover, the 100% relative activity was obtained without any additions as described by Shafiei et al. (2010)^31^.

### Fluorescence quenching analysis

The intrinsic fluorescence quenching assay was performed at 25 ℃ with an excitation wavelength of 280 nm^32^. The excitation and emission slit band widths were set at 3 and 5 nm, respectively. Briefly, 2.0 mL of α-amylase solution [0.04 mg/mL prepared with 50 mM HEPES pH 8] was mixed with 100 µL of acarbose at varying concentrations (15,30,45,60, and 75 mM). After 15 min of incubation at 50 ℃, the fluorescence emission spectra were recorded from 220 to 500 nm with a 1 cm Quartz cuvette. The fluorescence spectra of α-amylase solution read in the absence of acarbose (but with 100 µL of the buffer) under the same experimental conditions served as reference. The α-amylase fluorescence quenching mechanism in the presence of acarbose was analysed using the Stern-Volmer Eq. 1^32,33^. The binding constant (*K*_*a*_) and the number of binding sites (*n*) in α-amylase for acarbose were determined by the modified Stern-Volmer Eq. 2:

1 $$\:F0/F\:\:=1+Ksv\left[\text{Q}\right]$$

2 $$\:\text{L}\text{o}\text{g}\:(F0-F)/F=\:\text{L}\text{o}\text{g}\:Ka\:+n\text{L}\text{o}\text{g}\left[\text{Q}\right]$$

where *F0* and *F* represent the respective intrinsic fluorescence intensities in the absence and presence of acarbose [Q] is the concentration of acarbose, and *K*_*a*_ denotes the binding constant.

### End product analysis

Starch hydrolysis products were analysed by HPLC. 0.1 mL of each enzyme was incubated with 0.1 ml of 10% gelatinized starch in malate buffer (pH 6.5) at 50 °C for 16 h. Reactions were terminated by boiling for 10 min, cooled, and filtered through a 0.45 μm nylon syringe filter. Samples were separated on an aminopropyl column (4.6 × 250 mm, 5 µm particle size; 25046-5-SU-NH_2_) using acetonitrile–water (70:30, v/v) at 1 ml/min, 35 °C, and detected by refractive index. Glucose and maltooligosaccharides were served as standards.

### Statistical analysis

α-Amylase assays were carried out in parallel in triplicate sets. The values were determined and presented as the mean ± standard deviation. All statistical analyses were performed using GraphPad Prism 10.0^30^.

### Docking analysis and molecular dynamics simulation

The two-dimensional (2D) structure of acarbose was downloaded from the PubChem database (https://pubchem.ncbi.nlm.nih.gov/), converted to three-dimensional (3D) structures and thereafter prepared for docking using AutoDock Tools v1.5.6. Similarly, the 3D structures of BaqA∆C and BaqA∆CN185D were designed using alphafold2. Water from the X-ray structure was removed from the protein, followed by the addition of polar hydrogens before assignment of Kollman charges. A grid box size of 26 × 26 × 26 Å in the x, y and z dimension with 1.000 Å spacing in addition to grid box centers of 3.069, 2.399 and 1.556 Å (x, y and z) were used for the receptor-ligand docking interaction. AutoDock Vina software^34^ was used for the docking analysis and binding affinities generation, while molecular visualization of interacting amino acid residues at the protein active site was done by Edu PyMol (DLP 3D Viewer) and Ligplot^+^^35^. Molecular dynamics simulations were performed to explore the stability of α-amylase BaqA∆C and BaqA∆CN185D using AMBER 20 software^36^. The ff19SB force field was applied to set the parameters^37,38^. Model TIP3P water was added to the system box and was neutralized by the addition of Na^+^ ions along with water residues. The cutoff distance of 10 Å was used for calculations. The simulation began by minimizing the system’s energy consumption. The NVT ensemble was utilized for 300 ps to raise the temperature from 0 to 323.15 K. The temperature of the system was kept at 323.15 K, while the pressure was kept at 1 atm by utilizing a Langevin thermostat in conjunction with an isotropic position scaling method. The construction of the molecular dynamics simulation was performed for a total of 100 ns. Following the completion of the molecular dynamics simulation, the root-mean-square deviation (RMSD) and root-mean-square fluctuation (RMSF) parameters were examined with the use of the CPPTRAJ^39^. A secondary structure of protein was generated using PDBsum webserver^40^.

## Results

### Analysis of the conserved sequence regions of α-amylases

**Fig. 1:**
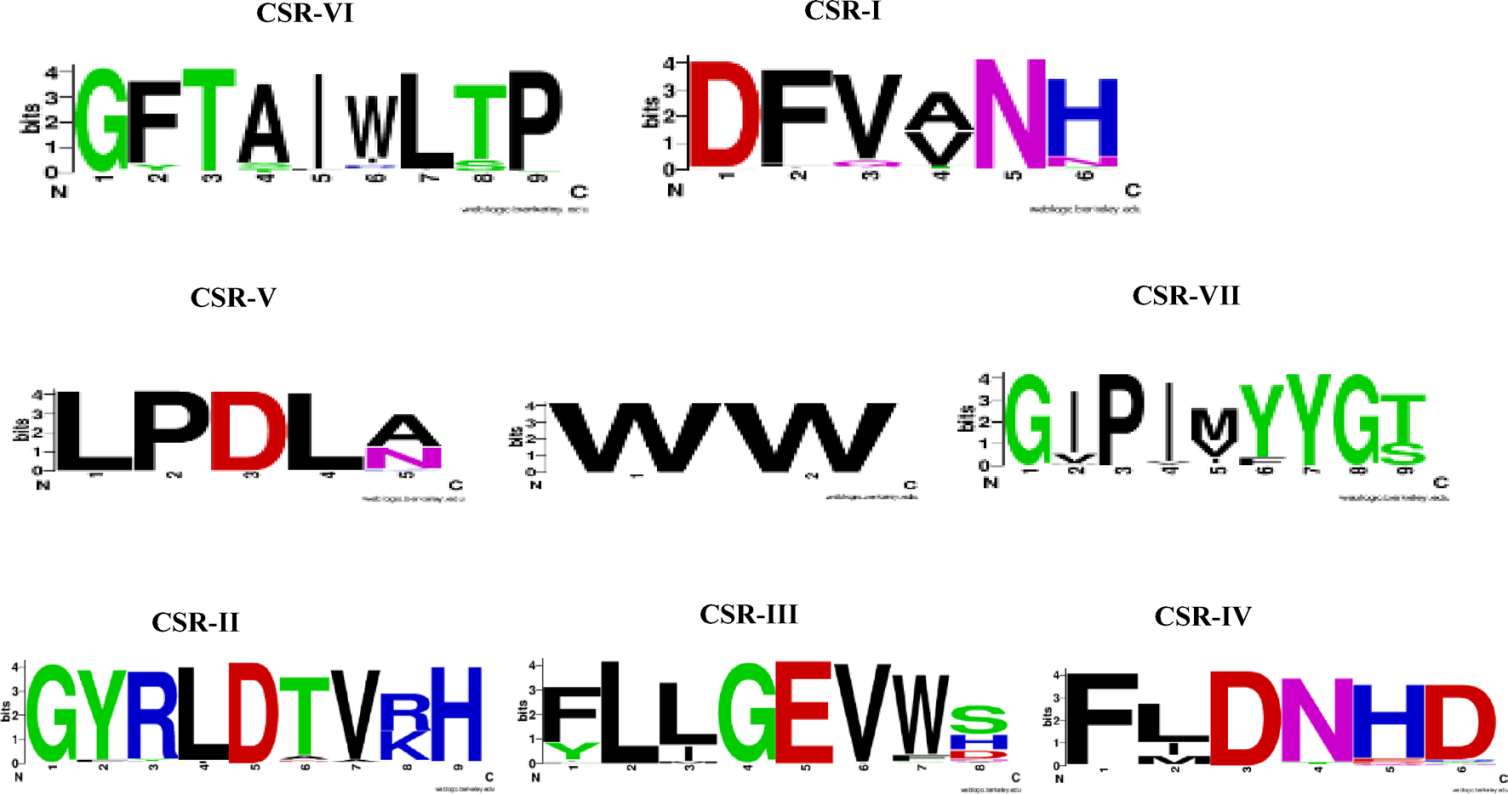
WebLogo of 140 α-amylases of the GH13_45 subfamily.

Until now, the majority of GH13_45 subfamily α-amylases remain uncharacterised. In this study, 140 α-amylase sequences were analysed, of which only twelve have been characterised (Figure S1), and seven conserved sequence regions (CSRs), characteristic of the GH13_45 subfamily, were identified, and their sequence conservation was visualized using WebLogo (Fig. 1).

**Fig. 2:**
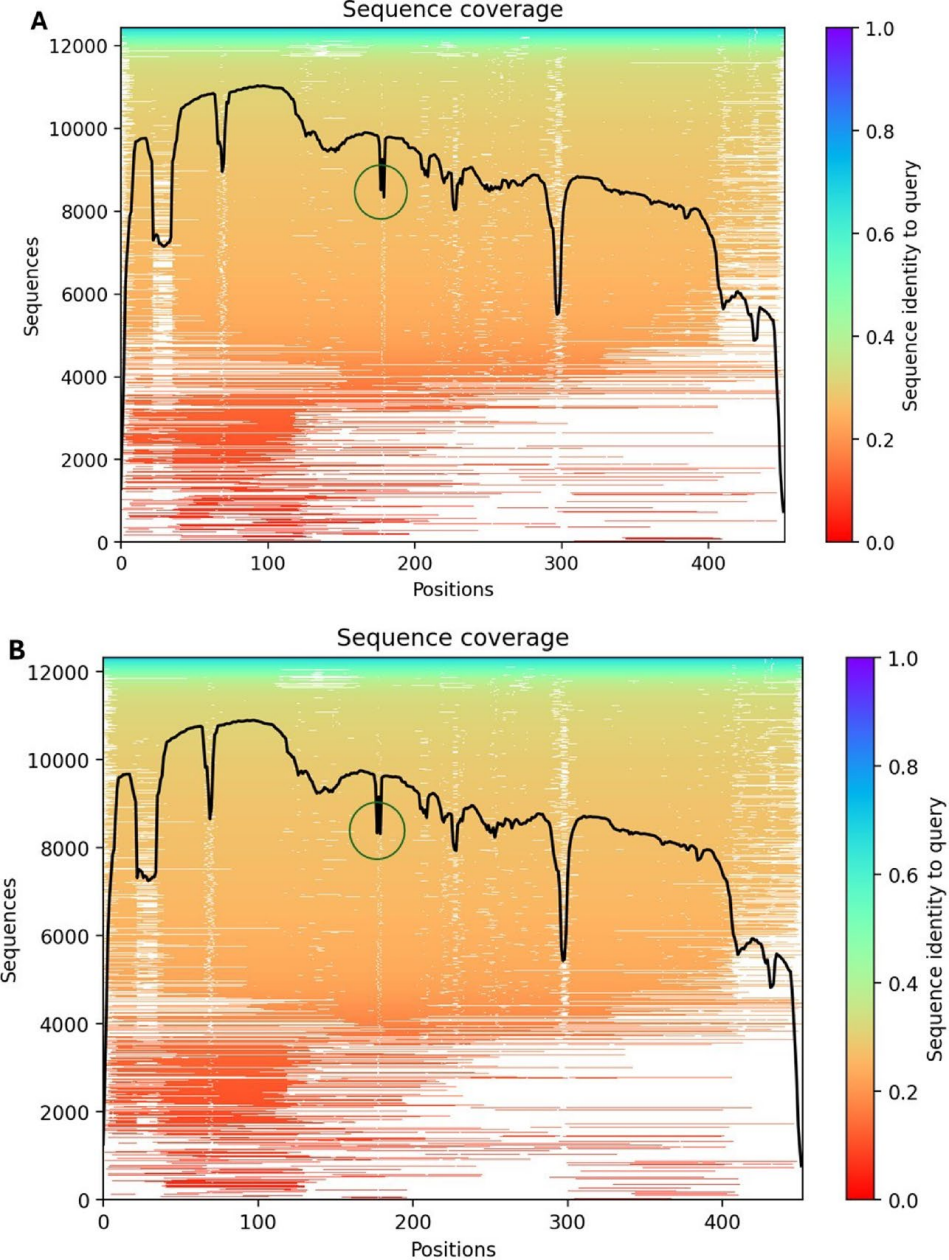
Illustration of sequence coverage plots (**A**) BaqAΔC, (**B**) BaqAΔCN185D.

BaqA∆C, along with its closely related characterised subfamily counterparts ASKA and ADTA, exhibited similar CSRs compared with other GH13_45 representatives. However, the majority of α-amylases within this subfamily lack complete CSRs. Sequence coverage plots from multiple sequence alignments (Fig. 2A and 2B) show that more than 8,000 homologous sequences cover most residues, suggesting strong evolutionary representation. High sequence identity (> 0.6) across many residues supports the robustness of the predicted structures. Localized drops in sequence coverage were observed in the vicinity of residues ~ 150, ~300, and ~ 420, which likely represent flexible or less conserved regions. The mutation site (highlighted) falls within the moderately conserved region, suggesting evolutionary tolerance for substitution.

**Fig. 3:**
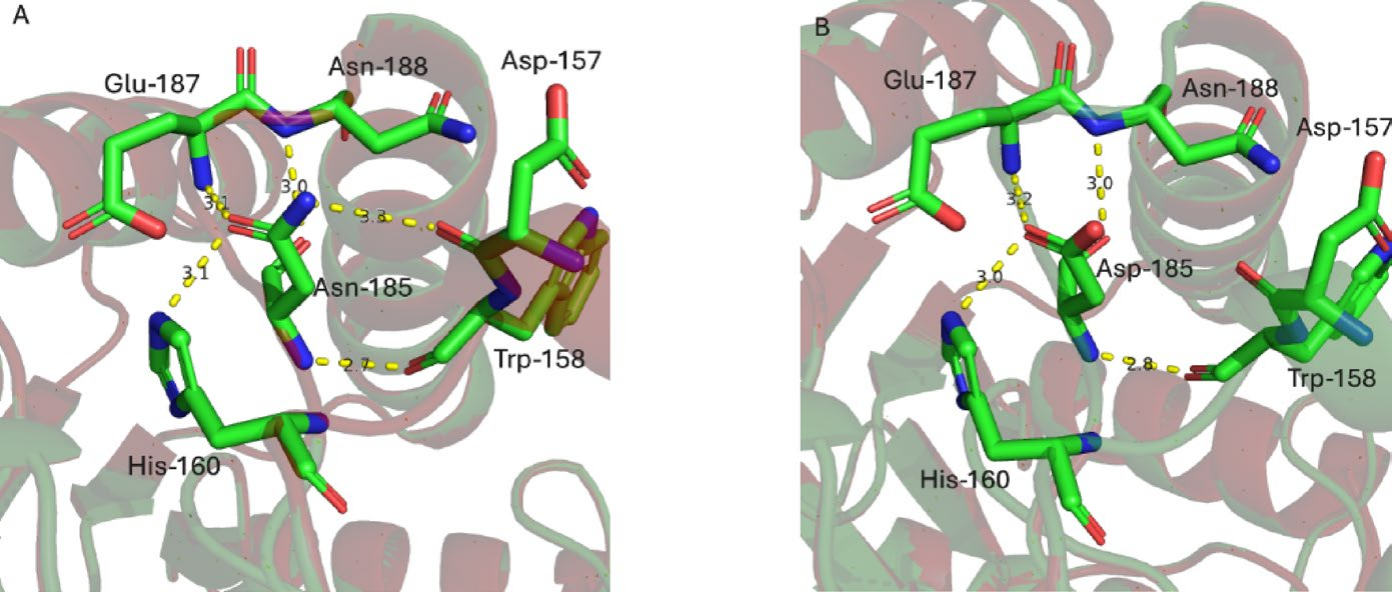
(**A**) Close-up view of the modelled structure of BaqAΔC, (**B**) Close-up view of the modelled structure of BaqAΔCN185D, changes introduced by mutation.

Three-dimensional structural models of both BaqAΔC and BaqAΔCN185D were predicted using ColabFold v1.5.5 and used for structural and comparative analyses (Fig. 3). In the BaqAΔC, residue Asn185 (N185) forms a network of five hydrogen bonds with surrounding residues, contributing to local stability. In contrast, substitution of N185 with aspartate in the BaqAΔCN185D reduces hydrogen bonding to four because of changes from amide (–CH₂–CONH₂) to carboxyl group (–CH₂–COOH). FoldX analysis^41^ indicated that the mutation had a stabilizing effect by lowering the protein total free energy.

### Expression and characterisation of the α-amylases

**Fig. 4:**
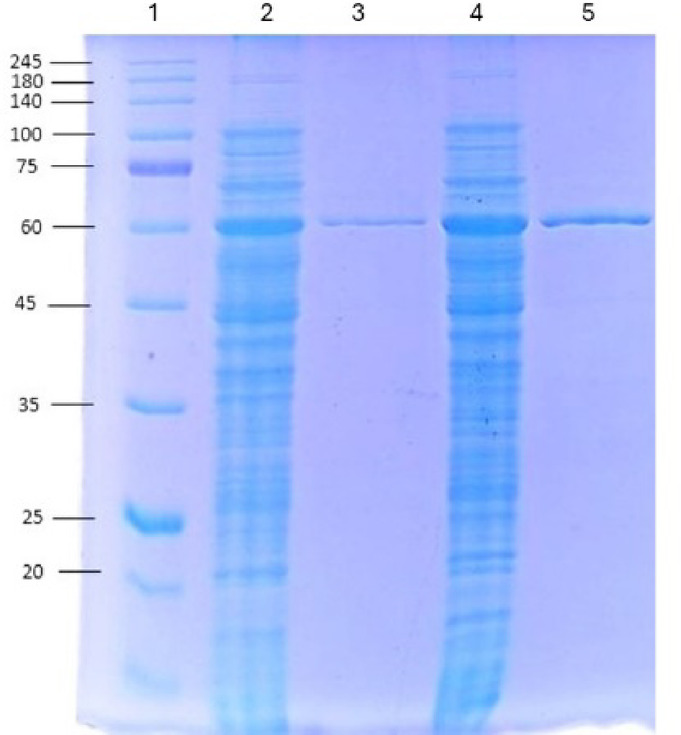
SDS PAGE analysis of BaqAΔC and BaqAΔCN185D. Lane 1: Protein marker; lane 2: crude extract of BaqAΔC; lane 3: purified BaqAΔC; lane 4: crude extract of BaqAΔCN185D, and lane 5: purified BaqAΔCN185D.

BaqAΔC and BaqAΔCN185D were expressed in *E. coli* ArcticExpress (DE3). SDS-PAGE analysis showed a protein of ~ 58.3 kDa, consistent with the predicted molecular weight. Both constructs contain an N-terminus (His)_6_ tag, enabling purification by Ni-NTA affinity column chromatography. BaqAΔC and BaqAΔCN185D were successfully purified to homogeneity, each appearing as a single protein band on SDS-PAGE (Fig. 4).

The specific activities of BaqA∆C and BaqA∆CN185D against 2% soluble starch were 5.8 ± 0.4 U/mg and 7.6 ± 0.2 U/mg, respectively. Remarkably, the degree of hydrolysis (DH) was 1.41% for BaqA∆C and 2.18% for BaqA∆C N185D, indicating a significant activity increase due to the mutation.

**Fig. 5:**
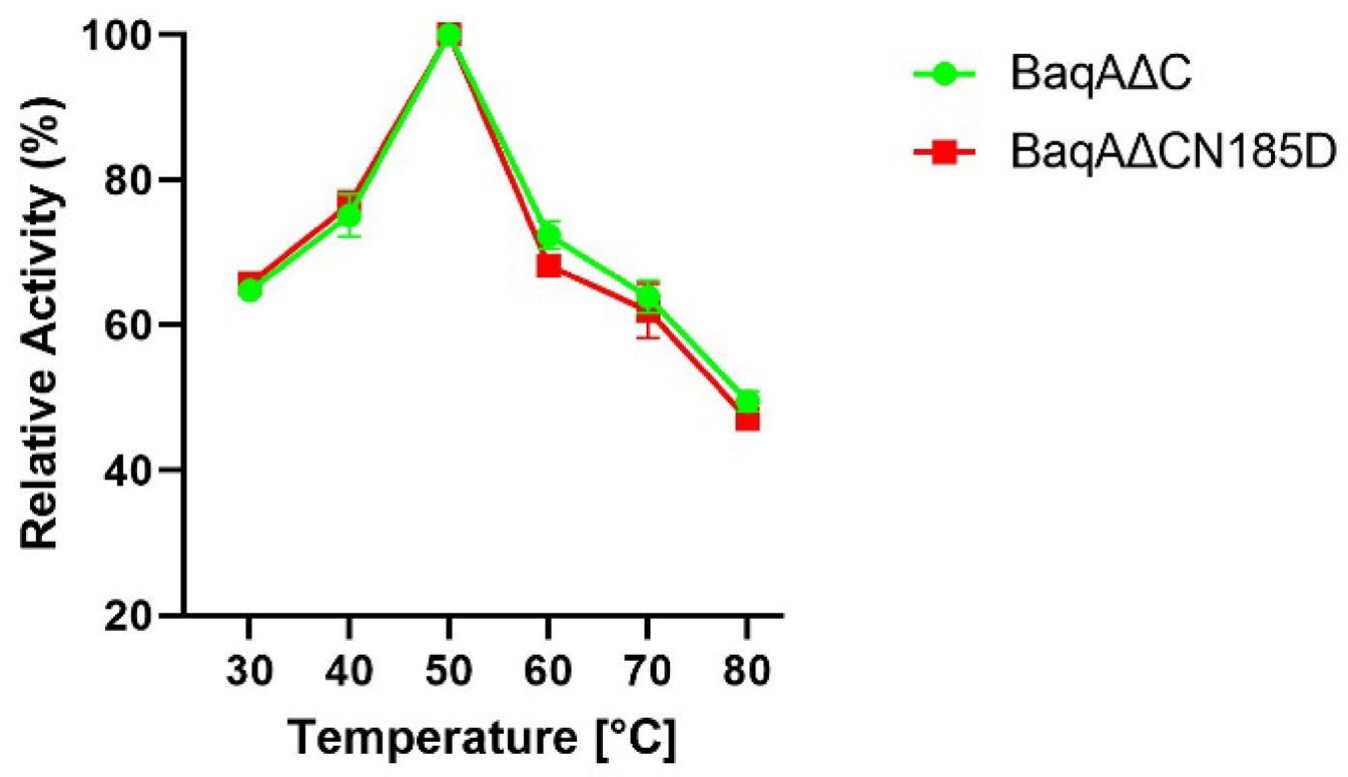
Effect of temperature on the activity of BaqAΔC and BaqAΔCN185D.

To investigate the effect of temperature on the activity, both enzymes were incubated with 2% (w/v) soluble starch across a temperature range of 30–80 °C at a pH of 6.5. Both BaqAΔC and BaqAΔCN185D exhibited optimal activity at 50 °C (Fig. 5) and retained approximately 50% of their activity, indicating that the N185D mutation had no significant effect on thermal stability.

**Fig. 6:**
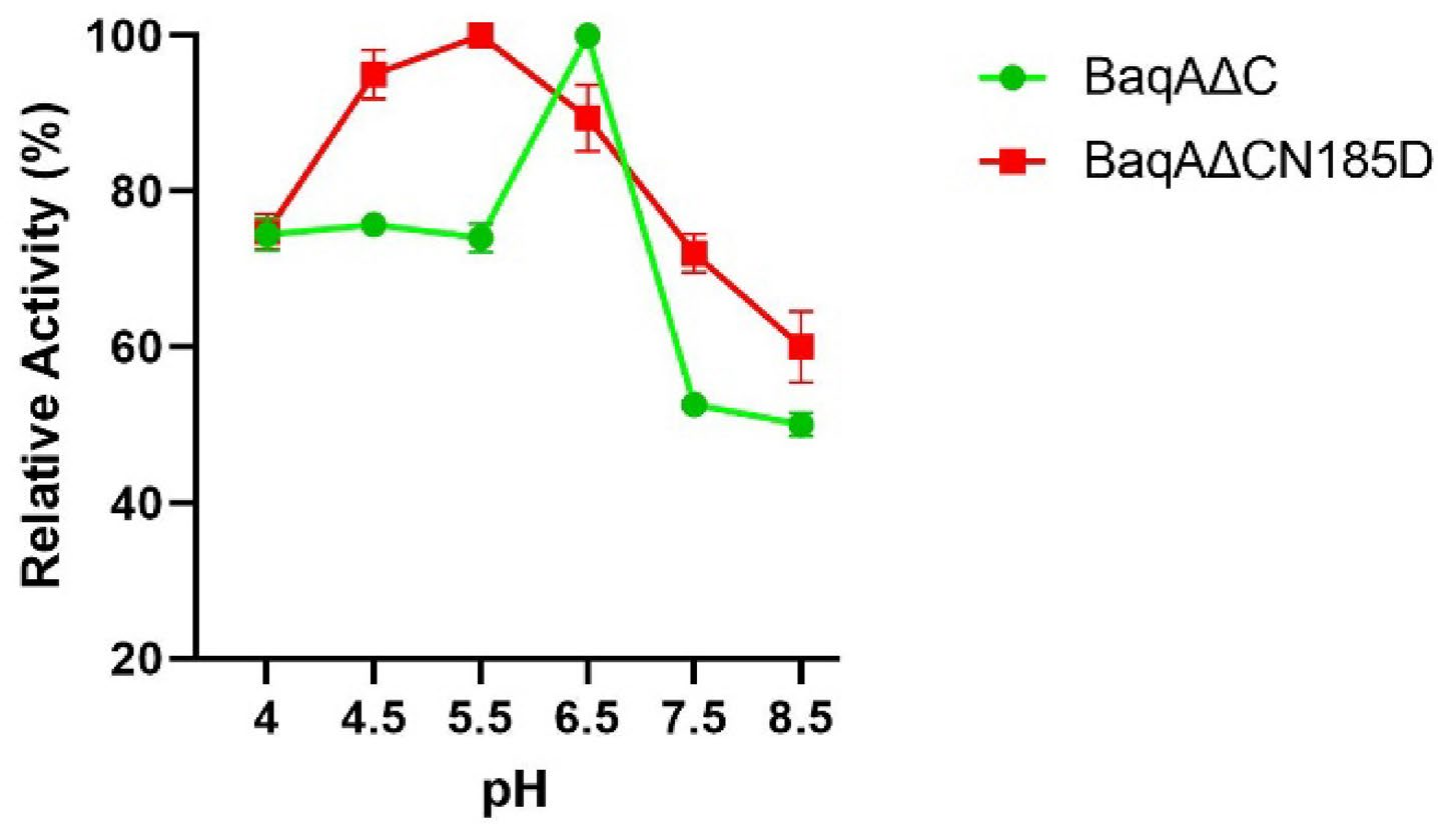
Effect of pH on the activity of BaqAΔC and BaqAΔCN185D.

The effect of pH on the activity of BaqAΔC and BaqAΔCN185D towards soluble starch was investigated by ranging the pH of the assay mixture from 4.0 to 8.5 at 50 °C. BaqAΔC showed optimal activity at a pH 6.5, whereas the mutant BaqAΔCN185D exhibited a higher optimal activity at pH 5.5. Notably, both enzymes retained more than 70% of their activity between pH 4.0 and 4.5. At higher pH values (6.6‒8.5), BaqAΔCN185D and BaqAΔC maintained 60% and 50% activity, respectively, indicating broader pH tolerance of the mutant compared to BaqAΔC (Fig. 6).

**Fig. 7:**
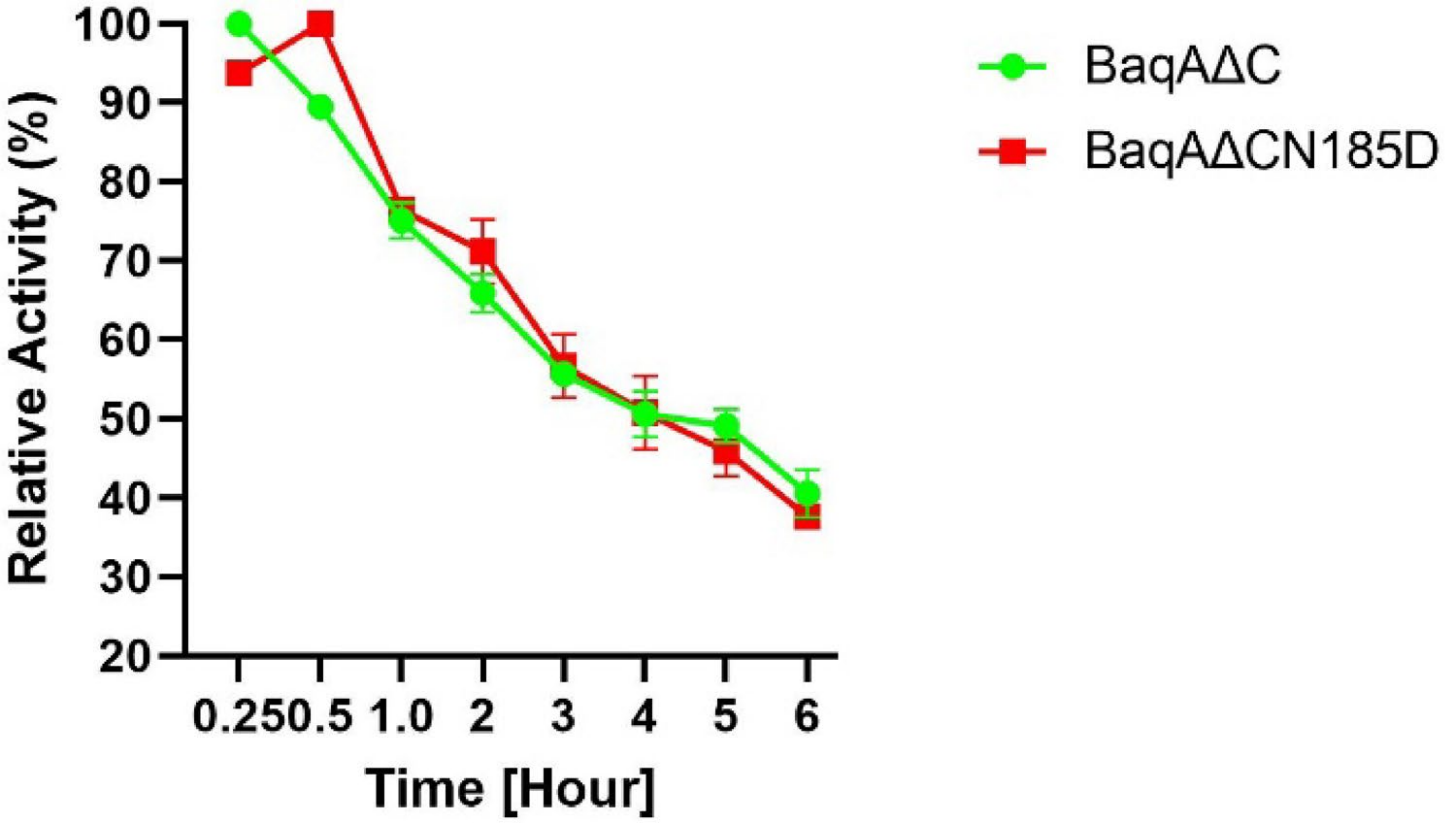
Thermostability profile of BaqAΔC and BaqAΔCN185D.

To confirm the thermostability of the enzymes, both BaqA∆C and BaqAΔCN185D were examined at their optimal temperature (50 ℃) and pH (6.5). The thermal inactivation profiles of BaqA∆C and BaqA∆CN185D were similar, suggesting that the thermostability of the enzyme remained unchanged upon mutation. Both BaqAΔC and BaqAΔCN185D retained approximately 40% of their activity after 6 h of incubation at 50 °C with a half-life of 4 h (Fig. 7).

To further investigate the basis of variation, kinetic parameters of BaqAΔC and BaqAΔCN185D were determined using a soluble starch as the substrate at optimal temperature (50 °C) and pH (6.5) (Table 1). Both enzymes followed the Michaelis-Menten kinetic profile (Figure S2). The mutant BaqAΔCN185D exhibited a markedly lower *K*_*m*_ (9.48 ± 1.3 mg/mL) than BaqAΔC (23.48 ± 4.8 mg/mL), indicating higher substrate affinity. The catalytic efficiency (*k*_*ca.*t_/*K*_*m*_) increased from 5.65 ± 0.5 to 9.8 ± 0.9 mL·mg⁻¹·s⁻¹. These results suggest that the N185D mutation enhances substrate binding and overall catalytic efficiency.

**Table 1:** Kinetic parameters of BaqA∆C and BaqA∆C N185D.

	BaqA∆C	BaqA∆C N185D
*K*_*m*_ (mg.mL^− 1^)	23.48 ± 4.8	9.48 ± 1.3
*k*_*cat*_ (s^− 1^)	137.7 ± 12.6	93.2 ± 4.9
*k*_*cat*_*/K*_*m*_ (mL.mg^− 1^.s^− 1^)	5.65 ± 0.5	9.8 ± 0.9

**Fig. 8:**
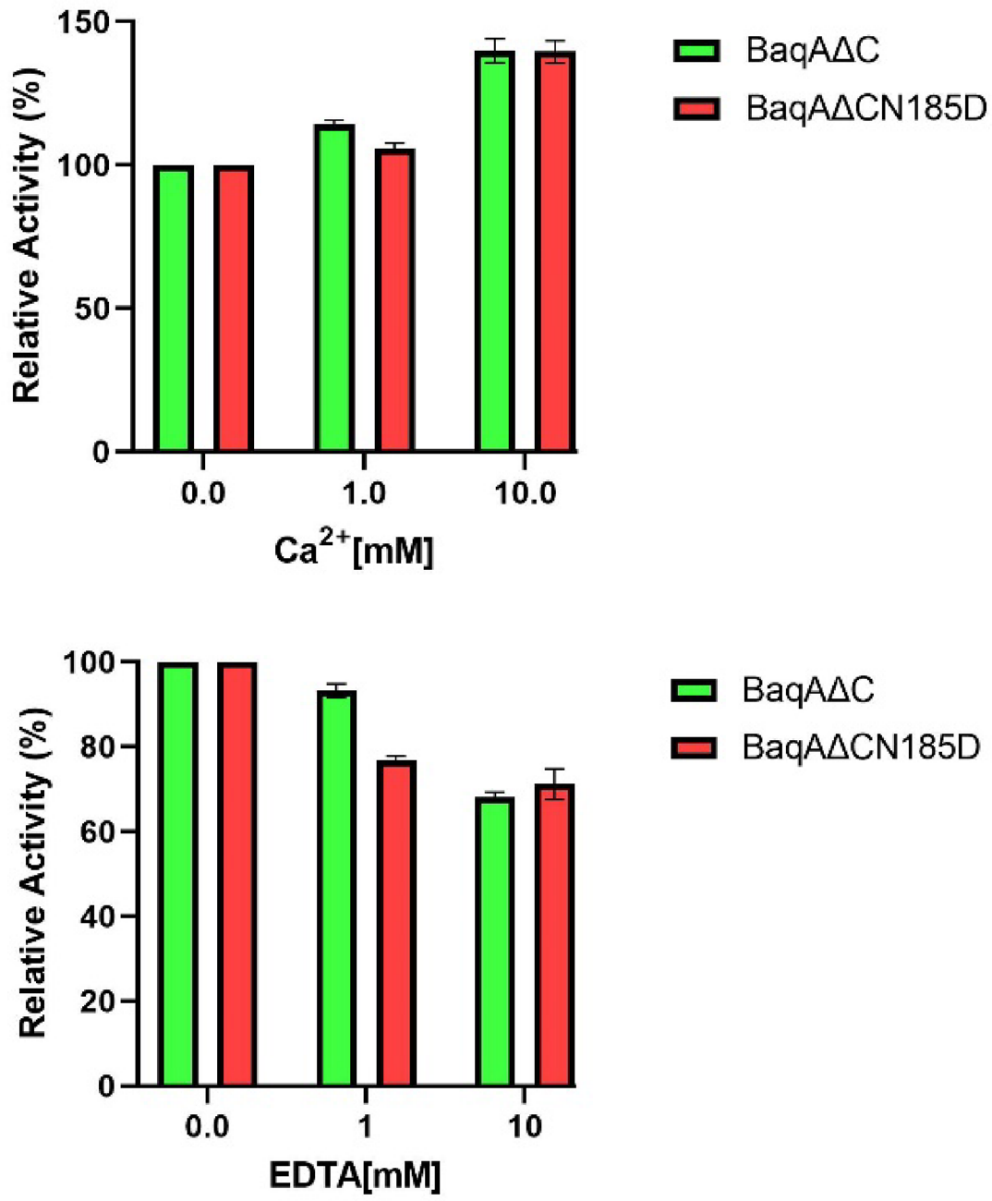
Effect of EDTA and Ca^2+^ on the activity of BaqAΔC and BaqAΔCN185D.

To analyse the effect of EDTA and [Ca^2+^] on enzyme activity, BaqAΔC and BaqAΔCN185D were pre-incubated with varying concentrations of EDTA and [Ca^2+^] (1–10 mM) at room temperature for 30 min. Interestingly, BaqAΔCN185D exhibited greater resistance towards EDTA as it retained 72% of its activity when incubated with 10 mM EDTA, while BaqAΔC retained 68% of its activity, which is quite higher compared to BmaN1∆C^42^. In contrast, ASKA and ADTA were completely inhibited upon the addition of 5 mM EDTA^43,44^. Surprisingly, both BaqAΔC and BaqAΔCN185D showed a 39% increase in activity upon treatment with [Ca^2+^] (Fig. 8), even though this activation was lower than that observed for ADTA (60%) and ASKA (99%) when treated with 5mM [Ca^2+^]^43,44^.

**Fig. 9:**
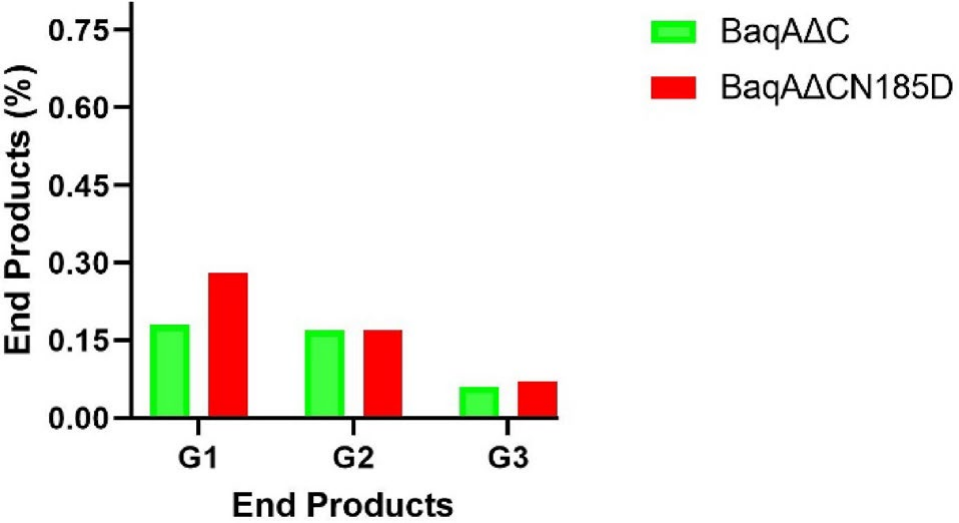
End products profile of BaqAΔC and BaqAΔCN185D activity. G1, G2 and G3 denote glucose, maltose and maltotriose respectively.

HPLC was used to analyse the starch hydrolysis products of crude extract from *E. coli* ArcticExpress (DE3) expressing BaqA∆C and BaqA∆CN185D. The control strain, lacking the target gene, produced a native liquefying α-amylase^45^, while BaqA∆C and BaqA∆CN185D exhibited saccharifying activity. Notably, BaqA∆CN185D yielded distinguished amount of glucose (Fig. 9). In contrast, α-amylase isolated from *B. licheniformis* SO-B3 yielded only glucose after 240 min of incubation^46^.

### Binding interaction analysis

**Fig. 10:**
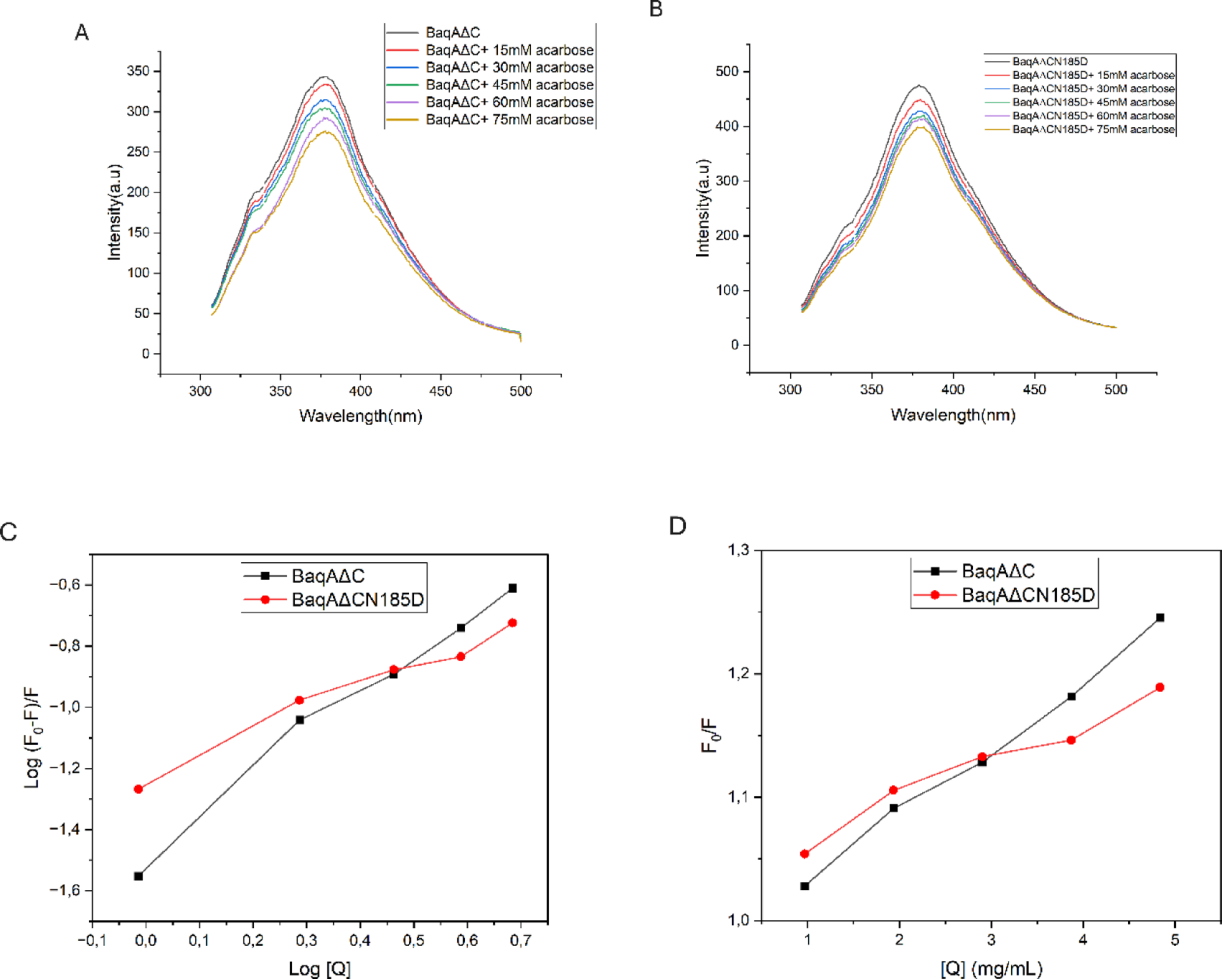
Quenching of α-amylase intrinsic fluorescence. (**A**) and (**B**) show the interaction between acarbose and BaqAΔC and BaqAΔCN185D, while (**C**) and (**D**) represent Intrinsic fluorescence spectra, Stern-Volmer (*F*_*0*_/*F* versus [Q]) and modified Stern-Volmer plots (Log (*F*_*0*_-*F*)/*F* versus Log [Q]) for α-amylase interaction with acarbose.

In this study, we measured the fluorescence emission intensities of BaqAΔC and BaqAΔCN185D at varying concentrations of acarbose and analysed the observed variations using Stern-Volmer and modified Stern-Volmer plots (Fig. 10) derived by using Origin Pro 9.1^47^. A Stern-Volmer plot of *F*_*0*_/*F* versus [Q] was used to determine the mode of fluorescence quenching, binding constant, and number of binding sites (Fig. 10). Additionally, a modified Stern-Volmer plot of Log [(*F*_*0*_-*F*)/*F*] versus Log [Q] was used to determine the binding constants (*K*_*a*_) and number of binding sites (*n*). The high binding affinity between the enzyme and acarbose was further supported by the binding constant values, with BaqAΔC and BaqAΔCN185D exhibiting *K*_a_ of 0.032 and 0.058 mL/mg, respectively.

Fluorescence spectral analysis revealed that the interaction between α-amylase and acarbose involved approximately a single binding site (*n* = 1.3 and 0.7 for BaqAΔC and BaqAΔCN185D, respectively), which is quite similar to the previously reported α-amylase from *Bacillus subtilis*^48^.

## Discussion

As of June 2025, CAZy classification system divides GH13 family into 49 subfamilies^8^. Individual amylases are often distinguished by unique sequence features in their CSRs, which may be associated with biochemical properties such as substrate binding, product specificity, and thermostability. The novel GH13_45 subfamily comprises 1091 α-amylases (CAZy; http://www.cazy.org/). Out of these, only 140 α-amylases meet the criteria proposed^22,49^ for inclusion in a new subfamily. BaqA has been classified within the GH13_45 subfamily. The sequence logo of GH13 subfamily 45 clearly illustrates that several residues are fully conserved across all seven conserved sequence regions (CSRs). Specifically, residues D, F, and N are conserved in CSR-I; G, Y, R, L, D, and H in CSR-II; L, G, E, and V in CSR-III; F, D, N, and D in CSR-IV; L, P, D, and L in CSR-V; G, T, I, L, and P in CSR-VI; and G, P, Y, and G in CSR-VII. This high level of conservation highlights their potential structural and functional significance within the subfamily.

In this study, we used a truncated form of recombinant BaqA^20^. A single point mutation was successfully carried out and expressed. The activity of BaqA∆CN185D was altered, resulting in a 1.5-fold increase in the presence of corn-starch. In maltose-producing α-amylases ASKA, ADTA^43^, Pizzo and GTA^12,50^, alanine is highly conserved at this identical position^21^. In Bst-MFA and BAA, substitution Asp→Asn, led to a decrease in enzyme activity, suggesting that this residue plays a role in enzymatic function^17,51^. While in ASKA, substitution of alanine with aspartate resulted in a two-fold increase in enzymatic activity^21^.

Both BaqAΔC and BaqAΔCN185D followed a similar amylolytic pattern when subjected to different temperatures. While ASKA and ADTA displayed an optimal temperature of 60 ℃^43^. At 70 ℃ to 80 ℃ BaqAΔC and BaqAΔCN185D retained more than 45% of their activity, whereas ASKA and ADTA maintained less than 40%^43^. This thermal variation is comparable to that of BmaN1∆C^42^ and suggest that BaqAΔC and BaqAΔCN185D exhibit broader temperature adaptability compared to GTA^12^. Furthermore, a similar substitution A161D at an identical position in ASKA did not alter its optimal temperature^21^.

The Asn→Asp mutation replaces a neutral polar residue with a negatively charged side chain. This alteration likely modifies the local electrostatic environment, perhaps influencing the pKa values of adjacent residues and adjusting the enzyme optimal protonation conditions. On the other hand, ASKA and ADTA retained only 20% of their activity at a pH of 4.0 and 60% at pH 5.0, with an optimal pH of 8.0, which is lower than the activity observed for BaqAΔC and BaqAΔCN185D (which retained more than 70% at pH 4–5)^43^. Meanwhile, GTA remained inactive at both pH 4.0 and 8.0^12^, highlighting the superior efficiency of BaqAΔCN185D in acidic conditions. BaqAΔCN185D exhibited a shift in optimum pH from 6.5 (BaqAΔC) to 5.5. According to Liu et al.. (2010)^51^, the mutation Asp→Asn caused pH instability due to decreased pH, while Lee et al.. (2006)^52^ reported that this mutation in BAA was associated with enhanced thermal stability.

Residue D185 is located at the surface of BaqAΔCN185D and it was hypothesized to influence the thermostability of the enzyme due to its presence near the calcium binding pocket. This outcome indicates that aspartate may not be essential in influencing thermal sensitivity or that the replacement with asparagine did not substantially disrupt the local structural environment. Similar observations have been reported previously where ASKA exhibited a half-life of 48 h while ADTA had a half-life of 3 h, despite sharing 98% amino acid sequence similarity with ASKA^43^.

The endogenous fluorescence intensities of α-amylases are associated with the presence of chromophoric groups, including tyrosine, tryptophan, and phenylalanine^53,54^. This implies that modifications to the microenvironments of specific amino acid residues can influence enzyme activity. To assess these variations, we evaluated changes in the maximum emission intensity of intrinsic fluorescence related to ligand-protein interactions^48^. BaqAΔC exhibited *K*_a_ of 0.032 and BaqAΔCN185D 0.058 mL/mg, respectively. On the other hand, BaqAΔCN185D exhibited catalytic efficiency (*k*_*cat*_*/K*_*m*_) of 9.8 ± 0.9 mL mg^− 1^ s^− 1^, which is higher than BaqAΔC *k*_*cat*_*/K*_*m*_ of (5.65 ± 0.5 mL mg^− 1^ s^− 1^). Both fluorescence analysis and kinetics support the increase in catalytic efficiency of BaqAΔCN185D. Similarly, the A161D mutation in ASKA (corresponding to N185D in BaqAΔC) resulted in a 2.88-fold increase in catalytic efficiency^21^.

**Fig. 11:**
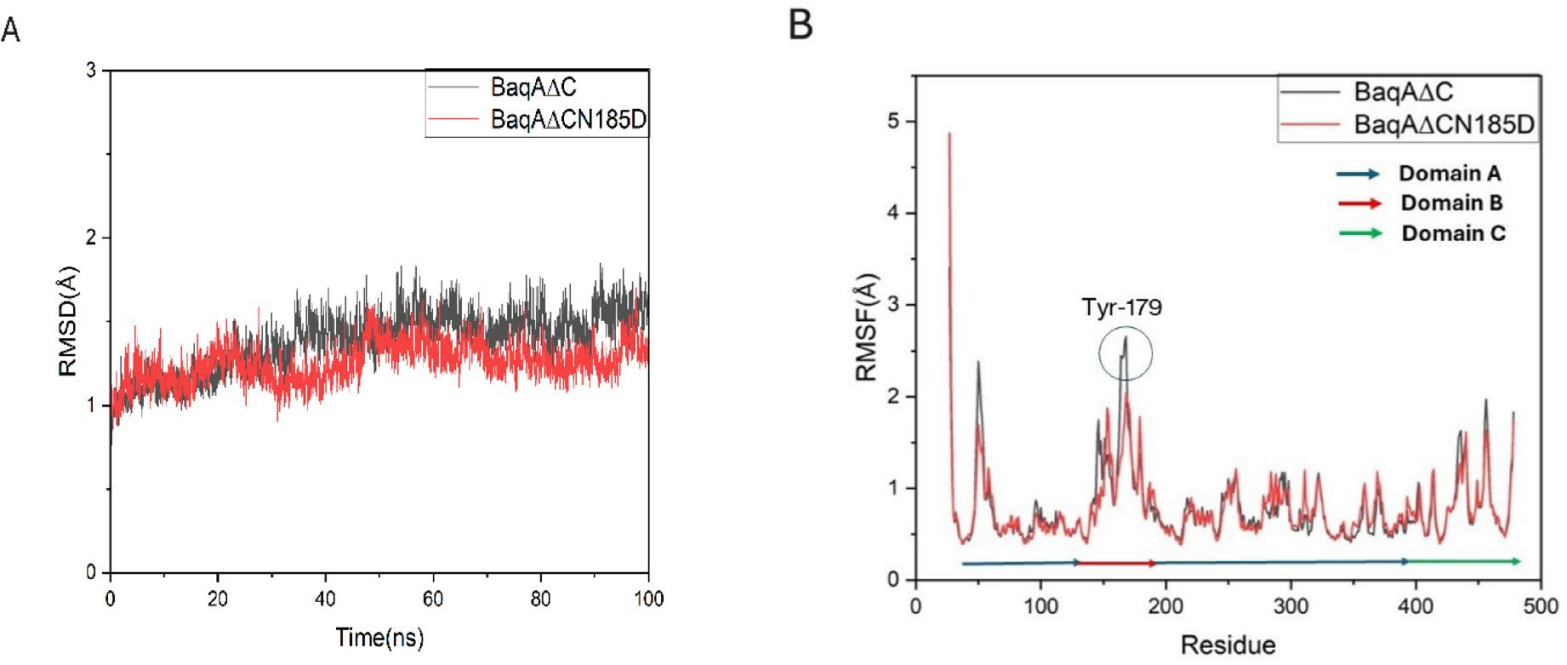
(**A**) RMSD profile of BaqAΔC and BaqAΔCN185D, (**B**) RMSF profile of BaqAΔC and BaqAΔCN185D.

Molecular docking analysis of acarbose with BaqAΔCN185D exhibited a binding affinity of − 7.8 (kcal/mol), which is quite higher than the binding affinity of BaqAΔC with acarbose (− 6.9 kcal/mol). RMSD analysis was used to track structural and conformational changes of the protein-ligand complexes. For each complex, the RMSD plot of protein-ligand complexes were computed for a 100-ns trajectory by using Origin Pro 9.1^47^. The plot showed RMSD value of BaqAΔC and BaqAΔCN185D to be 1.8 and 1.5 Å respectively (Fig. 11A), which is acceptable and indicates a stable system. While specific alterations as well as protein chain residues and variations in the position of the ligand atom at a certain temperature and pressure are analysed using the Root Mean Square Fluctuation (RMSF) (Fig. 11B). Furthermore, MM/PBSA^55^ calculations were employed to estimate the binding free energies of both BaqAΔC and BaqAΔCN185D with the substrate analog acarbose. The results revealed that BaqAΔCN185D exhibited a more negative Poisson-Boltzmann energy (−8.3 kcal/mol) compared to the BaqAΔC (0.1 kcal/mol), indicating a stronger interaction between BaqAΔCN185D and acarbose.

A more negative binding free energy reflects enhanced substrate binding affinity, which is typically associated with a lower *K*_*m*_. This is consistent with the kinetic data, where BaqAΔCN185D demonstrated a reduced *K*_*m*_ value, suggesting improved substrate recognition in the active site. Stronger binding can enhance the probability of enzyme–substrate complex formation, thereby increasing the rate of catalysis per unit substrate. The MM/PBSA results thus provide a thermodynamic basis for the enhanced binding and catalytic performance of the mutant, highlighting the structural impact of the point mutation on enzymatic function.

**Fig. 12:**
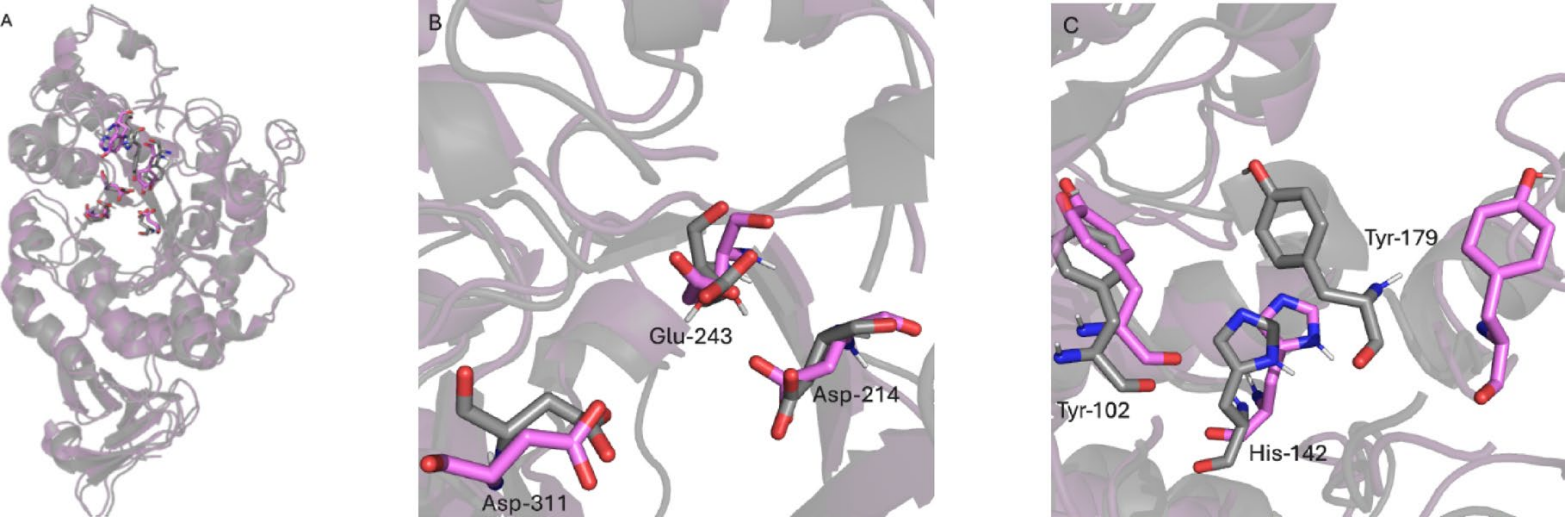
(**A**) Structural alignment of BaqA∆C and BaqA∆CN185D after MD, (**B**) Visualization of catalytic residues and (**C**) Visualization of predicted SBS residues, gray colour represents the BaqA∆C and light pink colour shows BaqA∆CN185D.

The MD simulation results reveal that mutation N185D causes changes in structure that may lead to enhancing the binding capacity and stability of BaqAΔCN185D with substrate (Fig. 12A, B and C). The MD simulation demonstrated that the structure of BaqA∆C was influenced by this substitution, causing comparable changes in catalytic and significant changes in predicted SBS residues (Tyr102, His142 and Tyr179)^56^, probably by providing a gateway to bind with the substrate more effectively. Furthermore, the two structures exhibit different residue arrangements that may alter the catalytic pocket. Such alterations in the catalytic pocket can lead to variations in the hydrolysis products.

## Conclusion

In this research, we compared the hydrolytic behaviour, pH stability, and catalytic efficiency of BaqA∆CN185D to that of BaqA∆C. The findings unequivocally showed that BaqA∆CN185D has increased catalytic efficiency, suggesting a possibly more effective enzymatic mechanism. Additionally, a wider pH range was demonstrated by the mutant enzyme, indicating its applicability for a variety of industrial and physiological conditions. Interestingly, the hydrolysis pattern of BaqA∆CN185D varied considerably from that BaqA∆C. The variation in hydrolysis products indicates an altered catalytic process, likely resulting from structural changes induced by the N185D mutation near the active site of BaqA∆C. These findings highlight the potential of BaqA∆CN185D for industrial applications requiring enhanced catalytic efficiency, stability, and specific saccharification profiles. However, further structural studies are necessary to fully elucidate the basis of these improvements and to guide future protein engineering efforts aimed at tailoring amylases for targeted biotechnological applications.

## Supplementary Information

Below is the link to the electronic supplementary material.

Supplementary Material 1

Supplementary Material 2

## Data Availability

The datasets generated and/or analyzed during the current study are available in the NCBI repository through the accession numbers provided in the text and supplementary data.
