# Supplementary material for: The role of N185D substitution in enhancing activity of BaqA∆C α-amylase
Source: Sci Rep. 2025 Dec 5;15:43521. doi: 10.1038/s41598-025-27398-8 (PMC12695923; doi:10.1038/s41598-025-27398-8)
Supplement: Supplementary file 1 — Supplementary Material 1 [file 41598_2025_27398_MOESM1_ESM.pdf]

The Role of N185D Substitution in Enhancing Activity of BaqAΔC α-Amylase

Muhammad Aqib Hanif [a], Reza Aditama [a], Ihsanawati [a], Fernita Puspasari [a], Dessy Natalia [a]*

[a] Biochemistry and Biomolecular Engineering Division, Faculty of Mathematics and Natural Sciences, Institut Teknologi Bandung, Jl. Ganesha No. 10, Bandung, Indonesia

*E-mail: dessynatalia@itb.ac.id

Supplementary data

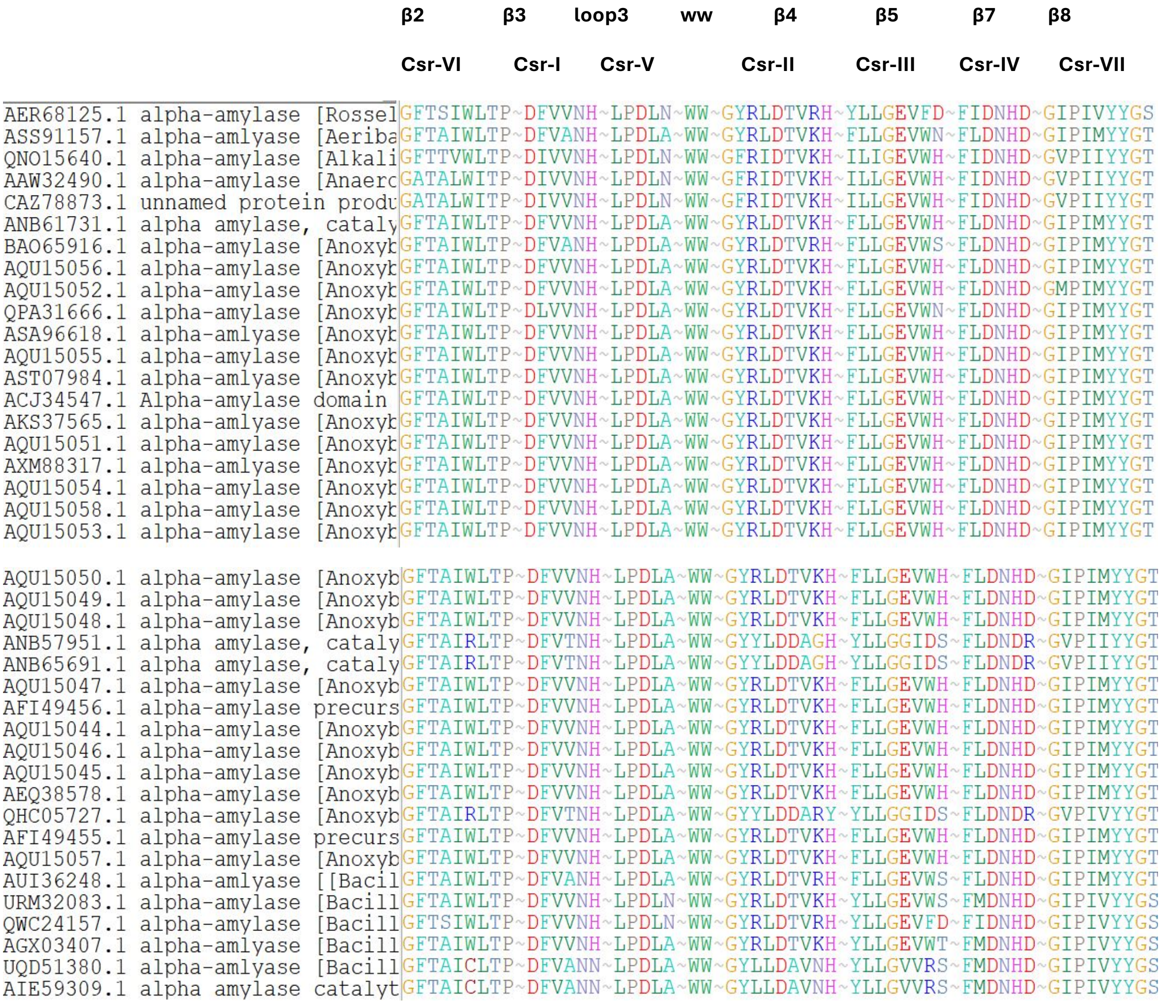

```
                                   β2        β3     loop3   ww    β4         β5        β7      β8
                                   Csr-VI    Csr-I  Csr-V         Csr-II     Csr-III   Csr-IV  Csr-VII

AGK52691.1 alpha amylase [Bacill   GFTAICLSP~EFPANS~LPDLN~WW~GYKLDHAAN~FLLGDIEA~FMDNQR~GIPIVYYGS
USK51204.1 alpha-amylase [Bacill   GFTAIWLTP~DFVVNH~LPDLK~WW~GYRLDTVKH~YLLGEVWS~FMDNHD~GIPIVYYGS
USK29590.1 alpha-amylase [Bacill   GFTSIWLTP~DFVVNH~LPDLN~WW~GYRLDTVRH~FLMGEVWD~FIDNHD~GMPIVYYGT
USK34759.1 alpha-amylase [Bacill   GFTSIWLTP~DFVVNH~LPDLN~WW~GYRLDTVRH~FLMGEVWD~FIDNHD~GIPIVYYGT
ALC91897.1 alpha-amlyase [Bacill   GFTAIMLSP~DFPANN~LPDLA~WW~GYRLSSADK~YLIADFGT~FMDHQD~GIPIVYYGS
ALC85618.1 alpha-amlyase [Bacill   GFTAIWLTP~DFVVNH~LPDLN~WW~GYRLDTVKH~FLIGEVWH~FLDTHD~GIPIVYYGT
AZB42321.1 alpha-amlyase [Bacill   GFTAIWLTP~DFVVNH~LPDLN~WW~GYRLDTVRH~FLLGEVWS~FLDNHD~GIPIVYYGS
UOY92110.1 alpha-amylase family   GFTAIWLTP~DFVGNH~LPDLN~WW~GFRLDAVKH~FLIGEVYN~YLDNHD~GIPIMYYGT
BCB03007.1 hypothetical protein   GFTAIWLTP~DFVVNH~LPDLN~WW~GYRLDTVRH~YLLGEVFD~FIDNHD~GIPIVYYGS
QGM32558.1 alpha-amylase [Bacill   GFTAIWLTP~DFVVNH~LPDLN~WW~GYRLDTVKH~YLIGEVWH~FIDNHD~GIPIIYYGT
QNG59629.1 alpha-amylase [Bacill   GFTSIWLTP~DFVVNH~LPDLN~WW~GYRLDTVRH~FLMGEVWD~FIDNHD~GMPVVYYGT
QCJ41730.1 alpha-amlyase [Bacill   GFTAIRLTP~DFVTNN~LPDLN~WW~GYSLPEVNH~FLLGVPTE~FFDNEY~GIPIFYYGT
QTC42088.1 alpha-amylase [Bacill   GFTSIWLTP~DFVVNH~LPDLN~WW~GYRLDTVRH~YLLGEVFD~FIDNHD~GIPIVYYGS
AYA75061.1 alpha-amlyase [Bacill   GFTAIWLTP~DFVVNH~LPDLN~WW~GYRLDTVRH~YLLGEVWD~FMDNHD~GIPIVYYGS
QOS88138.1 alpha-amylase [Brevib   GYTAIILSP~DFQANN~LPDLN~WW~GYRINKVQY~YTIGDVQG~FMDTQG~GVPIVFYGS
QYF81886.1 hypothetical protein   GYTAIILSP~DFQANN~LPDLN~WW~GYRINKVQY~YTIGDVQG~FMDTEG~GVPIVFYGS
QNK50703.1 alpha-amylase [Brevib   GYTAIILSP~DFQANN~LPDLN~WW~GYRINKVQY~YTIGDVQG~FMDTQE~GVPIVFYGS
QIB27253.1 alpha-amylase [Calora   GVTAIWITP~DYVVNH~LPDLN~WW~GMRLDTVKH~FLLGEVWN~FVDNHD~GIPIIYYGT
USK40237.1 alpha-amylase [Cytoba   GFTAIWLTP~DFVVNH~LPDLN~WW~GFRLDTVKH~YLLGEVWS~FMDNHD~GIPIMYYGS
UYG96850.1 alpha-amylase family   GFTAIWLTP~DFVVNH~LPDLN~WW~GYRLDTVKH~YLLGEVWS~FMDNHD~GIPIVYYGS

QKQ15316.1 amylase [Geobacillus    GFTALWVTP~EFVANH~LPDLA~WW~AYRLDTVRH~FLLCEVWS~FLDNHD~GIPIMYYGT
ACK58047.1 alpha-amylase [Geobac   GFTAIWLTP~DFVANH~LPDLA~WW~GYRLDTVRH~FLLGEVWS~FLDNHD~GIPIMYYGT
AMQ19772.1 alpha-amlyase [Geobac   GFTAIWLTP~DFVANH~LPDLA~WW~GYRLDTVRH~FLLGEVWS~FLDNHD~GIPIMYYGT
AKU27295.1 alpha-amlyase [Geobac   GFTAIWLTP~DFVANH~LPDLA~WW~GYRLDTVRH~FLLGEVWS~FLDNHD~GIPIMYYGT
ABL77406.1 alpha-amylase [Geobac   GFTAIWLTP~DFVANH~LPDLA~WW~GYRLDTVRH~FLLGEVWS~FLDNHD~GIPIMYYGT
BAP05653.1 amylase [Geobacillus    GFTAIWLTP~DFVANH~LPDLA~WW~GYRLDTVRH~FLLGEVWS~FLDNHD~GIPIMYYGT
ADU93127.1 alpha amylase catalyt   GFTAIWLTP~DFVANH~LPDLA~WW~GYRLDTVRH~FLLGEVWS~FLDNHD~GIPIMYYGT
ALA70997.1 alpha-amlyase [Geobac   GFTAIWLTP~DFVANH~LPDLA~WW~GYRLDTVRH~FLLGEVWS~FLDNHD~GIPIMYYGT
QHN48453.1 alpha-amlyase [Geobac   GFTAIWLTP~DFVANH~LPDLA~WW~GYRLDTVRH~FLLGEVWS~FLDNHD~GIPIMYYGT
QOR84891.1 alpha-amylase [Geobac   GFTAIWLTP~DFVANH~LPDLA~WW~GYRLDTVRH~FLLGEVWS~FLDNHD~GIPIMYYGT
ATA59064.1 alpha amylase catalyt   GFTAIWLTP~DFVANH~LPDLA~WW~GYRLDTVRH~FLLGEVWS~FLDNHD~GIPIMYYGT
QIZ66833.1 alpha-amylase [Geobac   GFTAIWLTP~DFVANH~LPDLA~WW~GYRLDTVRH~FLLGEVWS~FLDNHD~GIPIMYYGT
BBW97177.1 alpha-amylase [Geobac   GFTAIWLTP~DFVANH~LPDLA~WW~GYRLDTVRH~FLLGEVWS~FLDNHD~GIPIMYYGT
AMX84412.1 alpha-amlyase [Geobac   GFTAIWLTP~DFVANH~LPDLA~WW~GYRLDTVRH~FLLGEVWS~FLDNHD~GIPIMYYGT
AST00403.1 alpha-amlyase [Geobac   GFTAIWLTP~DFVANH~LPDLA~WW~GYRLDTVRH~FLLGEVWS~FLDNHD~GIPIMYYGT
ARA97568.1 alpha-amlyase [Geobac   GFTAIWLTP~DFVANH~LPDLA~WW~GYRLDTVRH~FLLGEVWS~FLDNHD~GIPIMYYGT
ABO65996.1 Alpha-amylase family   GFTAIWLTP~DFVANH~LPDLA~WW~GYRLDMVRH~FLLGEVWS~FLDNHD~GIPIMYYGT
ARP41727.1 Cyclomaltodextrinase   GFTAIWLTP~DFVANH~LPDLA~WW~GYRLDTVRH~FLLGEVWS~FLDNHD~GIPIMYYGT
QDY72399.1 alpha-amlyase [Geobac   GFTAIWLTP~DFVANH~LPDLA~WW~GYRLDTVRH~FLLGEVWS~FLDNHD~GIPIMYYGT
AEV18110.1 Alpha-amylase [Geobac   GFTAIWLTP~DFVANH~LPDLA~WW~GYRLDTVRH~FLLGEVWS~FLDNHD~GIPIMYYGT

URT72182.1 alpha-amylase family   GFTAIWLTP~DFVVNH~LPDLN~WW~GYRLDTVKH~YLLGEVWS~FMDNHD~GIPIVYYGS
QVY62845.1 alpha-amylase [Cytoba   GFTAIWLAP~DFVVNH~LPDLN~WW~GYRLDTVKH~YLLGEVWA~FMDNHD~GIPIVYYGS
AND38927.1 alpha-amlyase [Cytoba   GFTAIWLTP~DFVVNH~LPDLK~WW~GYRLDTVKH~YLLGEVWS~FMDNHD~GIPIVYYGS
UQX52227.1 alpha-amylase family   GFTAIWLTP~DFVVNH~LPDLK~WW~GYRLDTVKH~YLLGEVWS~FMDNHD~GIPIVYYGS
USK45710.1 alpha-amylase [Cytoba   GFTAIWLTP~DFVVNH~LPDLK~WW~GYRLDTVKH~YLLGEVWS~FMDNHD~GIPIVYYGS
UOE56715.1 alpha-amylase [Cytoba   GFTAIWLTP~DFVVNH~LPDLK~WW~GYRLDTVKH~YLLGEVWS~FMDNHD~GIPIVYYGS
QOK27483.1 alpha-amylase [Cytoba   GFTAIWLTP~DFVVNH~LPDLK~WW~GYRLDTVKH~YLLGEVWS~FMDNHD~GIPIVYYGS
QOR67521.1 alpha-glucosidase C-t   GFTSIWLTP~DFVVNH~LPDLA~WW~GYRLDTVRH~FLLGEVWH~FIDNHD~GIPIVYYGT
AGT31044.1 alpha-amlyase [Geobac   GFTAIWLTP~DFVANH~LPDLA~WW~GYRLDTVRH~FLLGEVWS~FLDNHD~GIPIMYYGT
BAD74992.1 alpha-amylase [Geobac   GFTAIWLTP~DFVANH~LPDLA~WW~GYRLDTVRH~FLLGEVWS~FLDNHD~GIPIMYYGT
CAZ78789.1 unnamed protein produ   GFTAIWLTP~DFVANH~LPDLA~WW~GYRLDTVRH~FLLGEVWS~FLDNHD~GIPIMYYGT
QCK82057.1 alpha-amlyase [Geobac   GFTAIWLTP~DFVANH~LPDLA~WW~GYRLDTVRH~FLLGEVWS~FLDNHD~GIPIMYYGT
ASS87985.1 alpha-amlyase [Geobac   GFTAIWLTP~DFVANH~LPDLA~WW~GYRLDTVRH~FLLGEVWS~FLDNHD~GIPIMYYGT
AKM17985.1 Beta/alpha-amylase pr   GFTAIWLTP~DFVANH~LPDLA~WW~GYRLDTVRH~FLLGEVWS~FLDNHD~GIPIMYYGT
QNU38157.1 alpha-amylase [Geobac   GFTAIWLTP~DFVANH~LPDLA~WW~GYRLDTVRH~FLIGEVWS~FLDNHD~GIPIMYYGT
QNU33656.1 alpha-amylase [Geobac   GFTAIWLTP~DFVANH~LPDLA~WW~GYRLDAVRH~LLLGEVWS~FLDNHD~GIPIMYYGT
QNU27301.1 alpha-amylase [Geobac   GFTAIWLTP~DFVANH~LPDLA~WW~GYRLDTVRH~FLLGEVWS~FLDNHD~GIPIMYYGT
QNU30448.1 alpha-amylase [Geobac   GFTAIWLTP~DFVANH~LPDLA~WW~GYRLDTVRH~FLLGEVWS~FLDNHD~GIPIMYYGT
ADI27796.1 alpha amylase catalyt   GFTAIWLTP~DFVANH~LPDLA~WW~GYRLDTVRH~FLLGEVWS~FLDNHD~GIPIMYYGT
AGE21200.1 maltogenic amylase [G   GFTAIWLTP~DFVANH~LPDLA~WW~GYRLDTVRH~FLLGEVWS~FLDNHD~GIPIMYYGT
```

	β2	β3	loop3	ww	β4	β5	β7	β8
	Csr-VI	Csr-I	Csr-V		Csr-II	Csr-III	Csr-IV	Csr-VII

AOL33596.1 alpha-amlyase [Geobac	GFTAIWLTP~DFVANH~LPDLA~WW~GYRLDTVRH~FLLGEVWS~FLDNHD~GIPIMYYGT
ATO36897.1 alpha-amlyase [Geobac	GFTAIWLTP~DFVANH~LPDLA~WW~GYRLDTVRH~FLLGEVWS~FLDNHD~GIPIMYYGT
AMV09992.1 alpha-amlyase [Geobac	GFTAIWLTP~DFVANH~LPDLA~WW~GYRLDTVRH~FLLGEVWS~FLDNHD~GIPIMYYGT
AFK08971.1 alpha-amylase II [Geo	GFTAIWLTP~DFVANH~LPDLA~WW~GYRLDTVRH~FLLGEVWS~FLDNHD~GIPIMYYGT
AWO73724.1 alpha-amlyase [Geobac	GFTAIWLTP~DFVANH~LPDLA~WW~GYRLDTVRH~FLLGEVWS~FLDNHD~GIPIMYYGT
ADG45817.1 alpha-amylase [Geobac	GFTAIWLTP~DFVANH~LPDLA~WW~GYRLDTVRH~FLLGEVWS~FLDNHD~GIPIMYYGT
QNU22814.1 alpha-amylase [Geobac	GFTAIWLTP~DFVANH~LPDLA~WW~GYRLDTVRH~FLLGEVWS~FLDNHD~GIPIMYYGT
UPT58563.1 alpha-amylase [Geobac	GFTAIWLTP~DFVANH~LPDLA~WW~GYRLDTVRH~FLLGEVWS~FLDNHD~GIPIMYYGT
QNU24175.1 alpha-amylase [Geobac	GFTAIWLTP~DFVANH~LPDLA~WW~GYRLDTVRH~FLLGEVWS~FLDNHD~GIPIMYYGT
QNU17998.1 alpha-amylase [Geobac	GFTAIWLTP~DFVANH~LPDLA~WW~GYRLDTVRH~FLLGEVWS~FLDNHD~GIPIMYYGT
AJD90706.1 hypothetical protein	GFTAIWLTP~DFVVNH~LPDLN~WW~GYRLDTVKH~YLIGEVYD~FIDNHD~GIPIMYYGS
QPC46047.1 alpha-amylase [Mangro	GFTAIWLTP~DFVVNH~LPDLA~WW~GYRLDTVKH~YLIGEVWS~FIDNHD~GIPIIYYGS
UYZ23171.1 alpha-amylase family	GFTAIWLTP~DFVVNH~LPDLA~WW~GYRLDTVRH~YLIGEVWS~FIDNHD~GIPIVYYGS
UAL53270.1 alpha-amylase [Metaba	GFTSIWLTP~DFVVNH~LPDLN~WW~GYRLDTVRH~FLMGEVWD~FIDNHD~GMPIVYYGT
QNF28381.1 alpha-amylase [Metaba	GFTTIALSS~EFVADH~LPDLA~WW~GYYIDHADT~YLLGSLLD~FIDSDN~GTPVVYYGS
ULT58112.1 alpha-amylase family	GFTAIRLTP~DFVANN~LPDLN~WW~GYSLPEVNQ~FLMGVPQD~FFDNEY~GIPIYYYGT
AZU61221.1 alpha-amlyase [Neobac	GFTTIRLTP~DFVTNN~LPDLN~WW~GYSLPEVNH~FLLGIPSK~FLDNEN~GIPVVYYGT
QAV26038.1 alpha-amlyase [Neobac	GFTAIWLTP~DFVVNH~LPDLA~WW~GYRLDTVKH~FLLGEVWH~FLDNHD~GIPIMYYGT
QXJ37790.1 Beta/alpha-amylase pr	GFTAIWLTP~DFVANH~LPDLA~WW~GYRLDTVRH~FLIGEVWS~FLDNHD~GIPIMYYGT
BDG34743.1 alpha-amylase [Parage	GFTAIWLTP~DFVANH~LPDLA~WW~GYRLDTVRH~FLIGEVWS~FLDNHD~GIPIMYYGT

BDG38517.1 alpha-amylase [Parage	GFTAIWLTP~DFVANH~LPDLA~WW~GYRLDTVRH~FLIGEVWS~FLDNHD~GIPIMYYGT
BDG42301.1 alpha-amylase [Parage	GFTAIWLTP~DFVANH~LPDLA~WW~GYRLDTVRH~FLIGEVWS~FLDNHD~GIPIMYYGT
BDG33249.1 alpha-amylase [Parage	GFTAIWLTP~DFVANH~LPDLA~WW~GYRLDTVRH~FLIGEVWS~FLDNHD~GIPIMYYGT
ALF09766.1 alpha-amlyase [Parage	GFTAIWLTP~DFVANH~LPDLA~WW~GYRLDTVRH~FLIGEVWS~FLDNHD~GIPIMYYGT
ANZ29847.1 alpha-amlyase [Parage	GFTAIWLTP~DFVANH~LPDLA~WW~GYRLDTVRH~FLIGEVWS~FLDNHD~GIPIMYYGT
APM80585.1 alpha-amlyase [Parage	GFTAIWLTP~DFVANH~LPDLA~WW~GYRLDTVRH~FLIGEVWS~FLDNHD~GIPIMYYGT
QIQ32749.1 alpha-amylase [Parage	GFTAIWLTP~DFVANH~LPDLA~WW~GYRLDAVRH~LLLGEVWA~FLDNHD~GIPIMYYGT
QSB48573.1 alpha-amylase [Parage	GFTAIWLTP~DFVANH~LPDLA~WW~GYRLDAVRH~LLLGEVWA~FLDNHD~GIPIMYYGT
USK60704.1 alpha-amylase [Periba	GFTTILLSP~DFVANS~LPDLN~WW~GYRLHQAEY~YLIGDVLE~FMDTSQ~GVPVIFYGS
USK71093.1 alpha-amylase [Periba	GFTTILLSP~DFVANS~LPDLN~WW~GYRLHQVEY~YLIGDVLE~FMDTSQ~GVPVIFYGS
USK86003.1 alpha-amylase [Periba	GFTTILLSP~DFVANS~LPDLN~WW~GYRLHQVEY~YLIGDVLE~FMDTSQ~GVPVIFYGS
AZV41653.1 alpha-amlyase [Periba	GFTTILLSP~DFVANS~LPDLN~WW~GYRLHQVEY~CLIGDVLE~FMDTSQ~GVPVIFYGS
QNU05952.1 alpha-amylase [Periba	GYTAIILSP~DFQANN~LPDLN~WW~GYRLNKVQY~YTIGDVQG~FMDTQN~GVPIVFYGS
AXN40159.1 alpha-amlyase [Periba	GYTAIILSP~DFQANN~LPDLN~WW~GYRLNKVQY~YTIGDVQG~FMDTQN~GVPIVFYGS
USK66350.1 alpha-amylase [Periba	GYTAIILSP~DFQANN~LPDLN~WW~GYRINKVQY~YTIGDVQG~FMDTQG~GVPIVFYGS
UZD48067.1 alpha-amylase family	GYTAIILSP~DFQANN~LPDLN~WW~GYRINKVQY~YTIGDVQG~FMDTQG~GVPIVFYGS
ULM98319.1 alpha-amylase family	GYTAIILSP~DFQANN~LPDLN~WW~GYRINKVQY~YTIGDVQG~FMDTQE~GVPIVFYGS
USK76130.1 alpha-amylase [Periba	GYTAIILSP~DFQANN~LPDLN~WW~GYRINKVQY~YTIGDVQG~FMDTQG~GVPIVFYGS
USK81512.1 alpha-amylase [Periba	GYTAIILSP~DFQANN~LPDLN~WW~GYRINKVQY~YTIGDVQG~FMDTQG~GVPIVFYGS
UYZ00138.1 alpha-amylase family	GYTAIILSP~DFQANN~LPDLN~WW~GYRINKVQY~YTIGDVQG~FMDTQG~GVPIVFYGS

AZV60915.1 alpha-amlyase [Periba	GYTAIILSP~DFQANN~LPDLN~WW~GYRINKVQY~YTIGDVQG~FMDTEG~GVPIVFYGS
AOH53884.1 alpha-amlyase [Periba	GFTAIILSP~DFQANN~LPDLN~WW~GYRLNKVQF~YTIGDIQG~FMDTQG~GVPIVFYGS
QQT02086.1 alpha-amylase [Periba	GFTAIWLTP~DFVVNH~LPDLN~WW~GYRLDTVKH~FLIGEVWD~FIDNHD~GIPIIYYGS
QFK70578.1 alpha-amylase [Prados	GFTTIALNS~DFNANH~LPDLA~WW~GYRLNDLEY~FLLGNGTI~MVDTED~GIPMVYYGS
UNL87130.1 alpha-amylase [Priest	GFTSIWLTP~DFVANH~LPDLN~WW~GYRLDTVRH~FLLGEVWN~FLDNHD~GIPIVYYGT
QIZ07088.1 alpha-amylase [Priest	GFTSIRLSP~DFVTNN~LPDLN~WW~GLSLPEVNY~FLLGVMSK~FMDNEY~GIPFFYYGS
QEY22869.1 alpha-amlyase [Psychr	GFTAIWLTP~DFVVNH~LPDLN~WW~GYRLDTVKH~YLIGEVWH~FIDNHD~GIPIIYYGT
QUG43694.1 alpha-glucosidase C-t	GFTAIWLTP~DFVVNH~LPDLD~WW~GYRLDTVKH~FLIGEVWH~FIDNHD~GIPIIYYGT
QHA35850.1 alpha-amylase [Rossel	GFTAIWLTP~DFVVNH~LPDLN~WW~GYRLDTVKH~YLLGEIFD~FIDNHD~GIPILYYGS
USK93775.1 alpha-amylase [Rossel	GFTAIWLTP~DFVVNH~LPDLN~WW~GYRLDTVKH~YLLGEIFD~FIDNHD~GIPILYYGS
UKS66825.1 alpha-amylase [Rossel	GFTAIWLTP~DFVVNH~LPDLN~WW~GYRLDTVKH~YLLGEVFD~FIDNHD~GIPILYYGS
UTE79109.1 alpha-amylase family	GFTAIWLTP~DFVVNH~LPDLN~WW~GYRLDTVRH~YLLGEVND~FIDNHD~GIPIVYYGS
QHE60968.1 alpha-amylase [Rossel	GFTAIWLTP~DFVVNH~LPDLN~WW~GYRLDTVRH~YLLGEVFD~FIDNHD~GIPIVYYGS
UXH45760.1 alpha-amylase family	GFTAIWLTP~DFVVNH~LPDLN~WW~GYRLDTVRH~YLLGEVFD~FIDNHD~GIPIVYYGS
AHC13589.1 Neopullulanase [Salin	GFSAIWISS~DLAINH~LPDLN~WW~GYRLDALRH~YLIGEVFD~FIDNHD~GIPIMYYGT
AST90565.1 alpha-amlyase [Sutcli	GFTAIWLTP~DLVVNH~LPDLN~WW~GYRLDTVKH~FLIGEVWD~FIDNHD~GIPIMYYGT
ART75769.1 alpha-amlyase [Sutcli	GFTALWLTP~DLVVNH~LPDLN~WW~GYRLDTVKH~FLIGEVWH~FLDNHD~GIPIMYYGT
WBL16215.1 alpha-amylase family	GFTAIWLTP~DLVVNH~LPDLN~WW~GYRLDTVKH~FLIGEVWD~FIDNHD~GIPIMYYGT
ALC91893.1 alpha-amlyase [Bacill	GFTAIWLTP~DFVVNH~LPDFN~WW~GYRLDTVRH~YLIGEVWT~FIDNHD~GIPIIYYGS
QFT88339.1 Beta/alpha-amylase pr	GFTALWLTP~DLVVNH~LPDIN~WW~GYRLDTVKH~FLIGEVWH~FLDNHD~GIPIIYYGT

Figure S1: CSR Sequence alignment of 160 α-amylases.
