# Supplementary figures and images for: The role of N185D substitution in enhancing activity of BaqA∆C α-amylase
Source: Sci Rep. 2025 Dec 5;15:43521. doi: 10.1038/s41598-025-27398-8 (PMC12695923; doi:10.1038/s41598-025-27398-8)

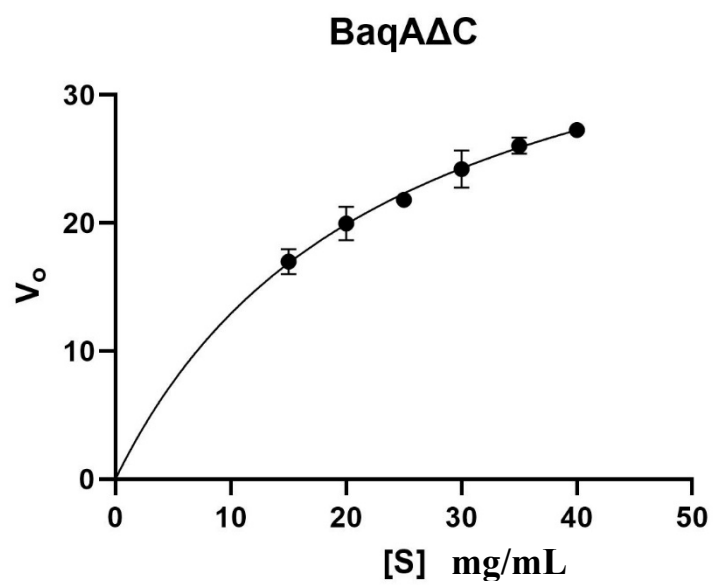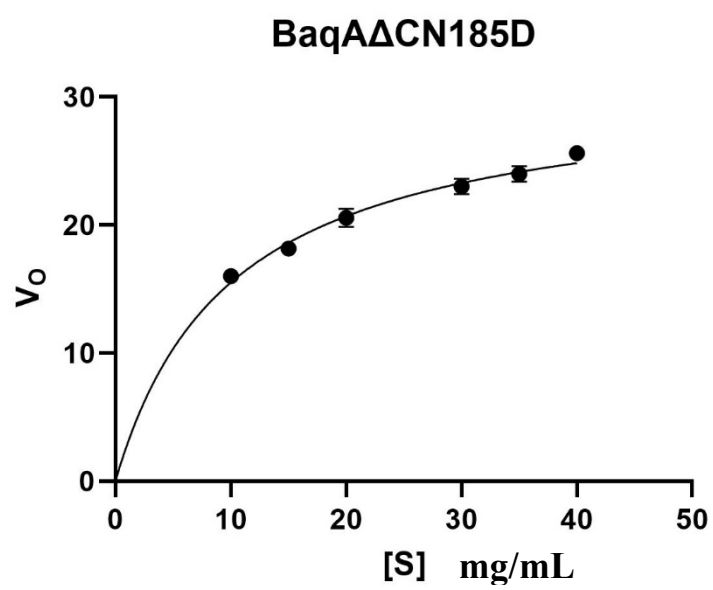

Supplement: Supplementary file 2 — Supplementary Material 2 [file 41598_2025_27398_MOESM2_ESM.pdf]
